# FogBank: a single cell segmentation across multiple cell lines and image modalities

**DOI:** 10.1186/s12859-014-0431-x

**Published:** 2014-12-30

**Authors:** Joe Chalfoun, Michael Majurski, Alden Dima, Christina Stuelten, Adele Peskin, Mary Brady

**Affiliations:** Information Technology Laboratory, National Institute of Standards and Technology, Gaithersburg, MD USA; Laboratory of Cellular and Molecular Biology, National Cancer Institute, National Institutes of Health, Bethesda, MD USA

**Keywords:** FogBank, Single cell segmentation, Robustness, Open-source

## Abstract

**Background:**

Many cell lines currently used in medical research, such as cancer cells or stem cells, grow in confluent sheets or colonies. The biology of individual cells provide valuable information, thus the separation of touching cells in these microscopy images is critical for counting, identification and measurement of individual cells. Over-segmentation of single cells continues to be a major problem for methods based on morphological watershed due to the high level of noise in microscopy cell images. There is a need for a new segmentation method that is robust over a wide variety of biological images and can accurately separate individual cells even in challenging datasets such as confluent sheets or colonies.

**Results:**

We present a new automated segmentation method called FogBank that accurately separates cells when confluent and touching each other. This technique is successfully applied to phase contrast, bright field, fluorescence microscopy and binary images. The method is based on morphological watershed principles with two new features to improve accuracy and minimize over-segmentation.

First, FogBank uses histogram binning to quantize pixel intensities which minimizes the image noise that causes over-segmentation. Second, FogBank uses a geodesic distance mask derived from raw images to detect the shapes of individual cells, in contrast to the more linear cell edges that other watershed-like algorithms produce.

We evaluated the segmentation accuracy against manually segmented datasets using two metrics. FogBank achieved segmentation accuracy on the order of 0.75 (1 being a perfect match). We compared our method with other available segmentation techniques in term of achieved performance over the reference data sets. FogBank outperformed all related algorithms. The accuracy has also been visually verified on data sets with 14 cell lines across 3 imaging modalities leading to 876 segmentation evaluation images.

**Conclusions:**

FogBank produces single cell segmentation from confluent cell sheets with high accuracy. It can be applied to microscopy images of multiple cell lines and a variety of imaging modalities. The code for the segmentation method is available as open-source and includes a Graphical User Interface for user friendly execution.

**Electronic supplementary material:**

The online version of this article (doi:10.1186/s12859-014-0431-x) contains supplementary material, which is available to authorized users.

## Background

Many cell lines that are currently being studied for medical purposes, such as cancer cell lines, grow in confluent sheets. These cell sheets typically exhibit cell line specific biological properties such as the morphology of the sheet, protein expression, proliferation rate, and invasive/metastatic potential. However, cell sheets are comprised of cells of different phenotypes. For example, individual cells in a sheet can have diverse migration patterns, cell shapes, can express different proteins, or differentiate differently. Identifying phenotypes of individual cells is highly desirable, as it will contribute to our understanding of biological phenomena of tumor metastasis, stem cell differentiation, or cell plasticity. Time-lapse microscopy now enables the observation of cell cultures over extended time periods and at high spatiotemporal resolution. Furthermore, it is now possible not only to label cells with fluorescent markers, but also to express fluorescently labeled protein, enabling spatiotemporal analysis of protein distribution in a cell sheet at a cellular level. To assess properties of individual cells within the observed sheet, however, it is necessary to accurately track these cells in a fully automated fashion. Thus, one of the requirements of an automated image analysis method is high accuracy single cell segmentation for individual time steps and its applicability to a wide range of cell types. Additionally, it is preferred that the developed method can analyze a multitude of image types, for example, phase contrast, differential interference contrast, and fluorescence images, as they are typically obtained in biomedical science.

Segmentation methods based on morphological watersheds are used for object separation and appear throughout the image processing and analysis literature and patents, since the method was first applied to image segmentation [1]. Most watershed methods work by dividing the image surface into regions based on pixel intensity gradient contours. However, the high level of noise in biological images leads to over-segmentation - a major problem when morphological watersheds are used [2-5]. This noise creates small minima across the regions of interest in an image, and gives rise to numerous small segmented regions that do not have biological significance. Therefore, a new segmentation method that accurately separates confluent cells into single cells for a wide range of applications is needed.

In general, watershed regions are formed either by a flooding process, expanding out from gradient minima, or by a watershed transform which computes a direct solution. Either of these methods can include the entire image, or begin from user-defined seed points. For flooding techniques, typically the regions are flooded according to intensity levels, through an immersion simulation [6] creating a topographic surface. Automatic minima detection can occur, for example, from low frequency components in the morphological gradient of an image [7]. Distance transforms can also be used for watershed segmentation, flooded from localized distance maxima [8]. Traditional watershed flooding by gradient level has been improved by adding local neighborhood comparisons and geodesic distance checking as the flooding occurs [9]. Gradient vector flow (GVF) [10], a diffusion of the classical gradient, has been used to give more weight to important feature edges. The viscous watershed technique [11] simulates flooding on a filtered relief of the image. More user-dependent methods extract regions through selected localized watershed flooding [12].

A variety of different watershed transforms are available, dating back from Meyer's watershed transform, which uses topographic distance to solve a shortest path function [11]. The Image Foresting Transform (IFT) [13] transforms an image into a weighted graph, in which each pixel is represented by a node in the graph. Cost functions are calculated for all possible paths within the graph to find the optimal region separation. The Tie-Zone Watershed (TZWS) transform [14] is derived from the IFT transform, and defines tie-zones, where regions overlap and the forests could produce multiple solutions, and defines unique optimal partitions between regions. Defining an energy minimization function to partition regions [15] more efficiently handles noisy images and incomplete boundaries, smoothing edges by adding a contour length to the energy function, and a locally constrained watershed transform [5] is based on such constraints. J. Cousty et al. [16] used Edge-weighted graphs to separate watershed basins, which are optimized using minimum spanning forests. Despite the long history of watershed techniques, to date none of these can successfully segment images of sheets of touching cells with high accuracy.

The task of separating watershed basins has been attempted in a number of ways, designed for specific types of cells. Merging criteria include region homogeneity and edge integrity [17], textures defined by co-occurrence matrices [18], distance transforms based on circular cell-like shapes [8], analyzing the gradient on multiple scales, hierarchical segmentation in which segmentation is a process ordered by decreasing altitude [19], and by flooding dynamics [20,21]. Local shape features from specific regions, extracted from Gaussian derivatives of the objects, are used to evolve region boundaries [15]. Spurious minima points have been merged according to an overlap parameter that measures the fractional overlap when the objects are treated as overlapping circles [3]. Graph segmentation has been used to find skeletal lines representing cell shapes for round and ellipsoidal objects [22,23], and the shape of segmented masks themselves used to separate circular objects [24,25]. The use of the Maximally Stable Extremal Regions (MSER) for edge detection followed by Ultimate erosion, watershedding, and fragment merging pipeline is used on bright field images [26]. All of these techniques are specific to one cell line or one image modality and require an expensive merging criterion that does not produce accurate results when applied to a different type of cell line or image modalities.

We present a new algorithm that can address the need for high accuracy single cell segmentation in confluent sheets or clusters of cells touching each other, and that can be applied to multiple cell lines and image modalities. We have developed a derivative improved watershed algorithm that automatically detects distinct basins (seed points) while minimizing over-segmentation and uses geodesic distances to preserves the shape of individual cells. It uses two methods for the reliable seed detection: (1) histogram quantization with seed size constraint, and (2) nucleoli seed detection, which incorporates biological insight to locate cell nuclei and their clustering. Furthermore, in the literature, the geodesic distance is mainly used to compute the shortest path between two points of interest while avoiding obstacles in the image [5,27,28]. In our method, we use the geodesic distance to assign pixels to the closest seed point object in the image which leads to individual cell shapes close to manually drawn ones, in contrast to the more linear cell edges that other watershed-like algorithms produce. We show that our new algorithm produces segmentation accuracy on 109 reference images in the order of 0.75, more successfully than previous methods. We compared our results to five freely available tools that worked on our reference datasets: CellProfiler based on region growing [29], CellTracer [30], Schnitzcells [31], Frlbm using level sets [32], and Marker-Controlled Watershed (MCW). We highlight the major differences between our new approach and previously existing ones and show its efficiency on a wide variety of applications. We visually verified our method on datasets comprised of 3 image modalities, 14 cell lines for a total of 876 images.

Section Methods describes our new method. In section Results, we quantify our results with this new method and compare our method to others. We also demonstrate the algorithm on multiple image modalities and cell lines. Section Discussion and Conclusion are dedicated to discussing the results and deriving conclusions.

## Methods

The automated single cell segmentation algorithm is comprised of five steps:

1. Separate foreground from background, defining the Region of Interest (ROI)
2. Detect potential cell boundaries in the image that will be used as barriers in the computation of the geodesic distance mask
3. Detect seed points or distinct basins within the ROIs
4. Separate single cell boundaries within the ROIs using seed points and boundary masks applied on modified grayscale images
5. Detect mitotic cells and add them to the mask

The following subsections describe each of the five algorithmic steps in detail.

### Foreground-background separation

We begin the process of separating a sheet of cells by locating the boundaries of that sheet using the Empirical Gradient Threshold (EGT) technique [29]. A gradient image is formed from the original image, and the foreground and background distributions of gradient magnitude values are separated based on their overlap. This technique has also been found to be highly accurate across imaging modalities and with a wide range of cell lines.

**Figure 1:**
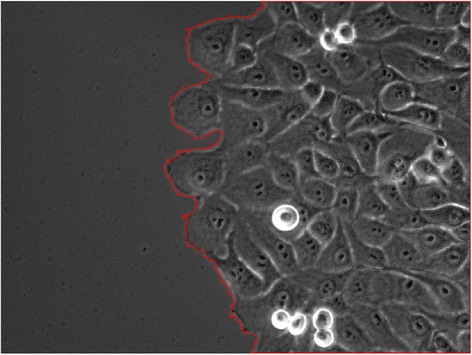
**Edge segmentation results.** Segmentation of sheet edges overlaid on the phase contrast image. The red color represents the colony edge and it is used only for highlight.

Figure 1 is an example of edge detection on an image of a sheet of breast epithelial cells. For more information about breast epithelial cells, please refer to [30,31].

### Geodesic distance and cell boundaries

The geodesic distance *d*_*I*_ (*a, b*) between two pixels *a* and *b* in the image *I*, as defined in [32], is the minimum of the length *L* of the path(s) *P* = (c_1_,c_2_,…,c_1_) joining *p* and *q* in *I*.

$$ \begin{array}{l}{d}_I\left(a,b\right)= \min\;\left\{L(P)\left|{c}_1\right.=a,{c}_1=b,P\subseteq I\right\}\hfill \\ {}{d}_I\left(a,b\right)=\infty, \kern0.3em \mathrm{if}\kern0.3em a\kern0.3em \mathrm{and}\kern0.3em b\kern0.3em \mathrm{are}\ \mathrm{not}\ \mathrm{connected}\kern0.3em \mathrm{in}\kern0.3em I.\hfill \end{array} $$

**Figure 2:**
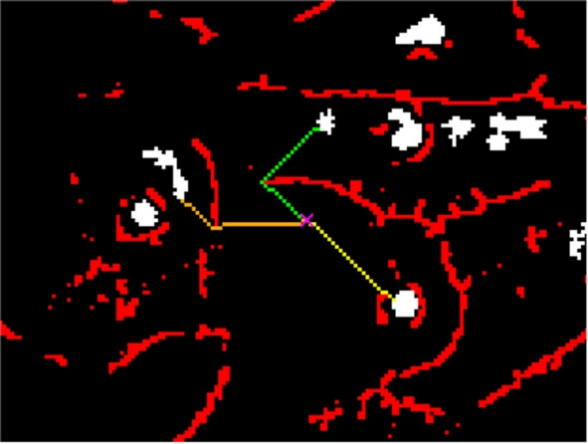
**Geodesic distance illustration.** A schematic figure to display the allocation of an unassigned pixel (x marked) to the closest seed point (yellow path) by means of the minimum geodesic distance between that pixel and the seed points in the image. The yellow path has a geodesic distance smaller than the orange or green path. The red pixels represent cell boundaries that cannot be traversed.

The geodesic distance prevents pixels that are close to a cell but separated by a boundary from being assigned to that cell. Those pixels are instead assigned to a different cell that is further away in terms of number of pixels on the image, but closer in terms of geodesic distance as shown in Figure 2.

**Figure 3:**
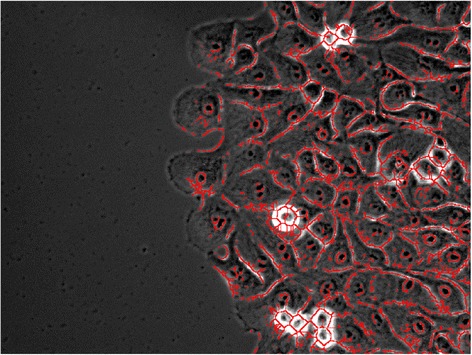
**Geodesic mask.** Geodesic mask that defines boundaries that cannot be traversed between cells highlighted in red.

There are two choices to define the boundary mask: (1) all pixels can be traversed, or (2) the geodesic mask is used. The geodesic mask [32] is a binary image where pixels with value equal to zero represent boundaries that cannot be traversed, and pixels with value equal to one are paths that can connect two pixels of interest together. Figure 3 shows the geodesic mask overlaid on the original phase image where the red pixels are the boundaries that cannot be traversed. Boundaries are defined through a user input percentile threshold, where the boundaries are considered to have high pixel intensities. In our case, the boundaries are composed of pixels with intensities higher than the 85th percentile intensity. This mask can help separate single cells with boundaries close to the ones drawn manually.

### Spatial seed point detection

The detection of seed points determines whether an image is over or under-segmented. Commonly used watershed-derived methods tend to lead to over-segmentation. This problem can be fixed by post processing steps that re-attach broken cell segments. These steps are challenging and lead to lower accuracy in the resulting images [22].

In our approach, in contrast to most watershed approaches, we operate on the image histogram or on the corresponding gradient histogram. We have developed two different methods for automatic detection of seed points that minimize over-segmentation: (1) histogram percentile binning quantization with seed size constraint, which does not incorporate any biological modeling, and (2) nucleoli seed detection, which incorporates biological insight to locate cell nuclei and their clustering. The user can choose either of these two methods prior to the automatic seed detection. The choice depends on the problem being solved. Examples showing advantages of each technique are presented in the Additional file 1.

#### Histogram quantization with seed size constraint

**Figure 4:**
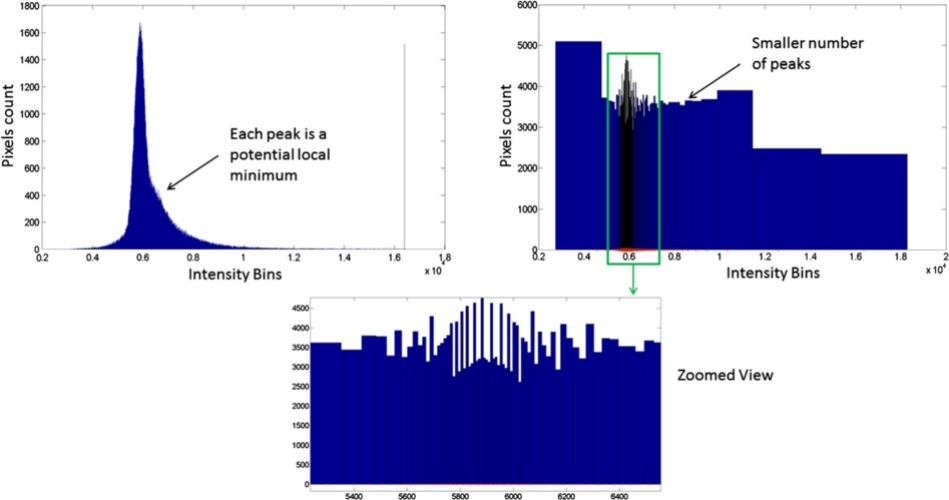
**Histogram quantization.** Image histogram with every pixel frequency displayed (top left), every bin contains a unique intensity value. Percentile binned histogram (top right and bottom): every bin contains 1% of the intensity values. Potential local minima correspond to peak values in the histogram where the corresponding intensity/location in the image might be considered as a seed point. Histogram quantization minimizes the number of local minima in the image, thus reducing the chances of over-segmenting the image.

This computational step computes seed points as a function of histogram percentile binning quantization with seed size constraint. In contrast to other techniques, intensity thresholds are not defined at every unique intensity value in the image but rather at each percentile value of the image. Using every unique value leads to multiple local peaks and hence to over-segmentation, while binning the pixel intensities reduces over-segmentation. For our purposes we used bins containing 1% of pixels. An illustration of the corresponding intensity interval is shown in Figure 4. The quantization reduces the number of potential seed points to consider, thus reducing the chances of over-segmenting the image. Furthermore, the use of percentiles helps to focus on the intensity levels that are more consistent across each quantile, and has a much faster execution time since we are considering only 100 intensity levels in any image. Figure 4 shows that the intensity levels are more concentrated in the middle section of the histogram and less on the boundaries.

Depending on image modality and cell line (e.g., phase contrast, fluorescent, binary with distance transform, etc.) one may want to look at seed points starting from low intensity moving to high intensity or vice versa. The two cases are color-coded below: (1) high intensity pixels correspond to seed points and low intensity pixels correspond to boundaries (in blue), and (2) low intensity pixels correspond to seed points (in red):

• The histogram *H* of an image is binned into 100 bins centered on the percentile values *p*(*i*) of the image. *p*(*i*) is the intensity value such that *i%* of image pixels have intensities less than *p*(*i*).
• Quantization is performed on every percentile level, starting from *p*(100) or *p*(*1*),
  - ○ Compute binary mask BW: *BW* = *I > p*(*i*) or *BW* = *I > p*(*i*),
  - ○ Apply pixel connectivity analysis to label the current mask,
  - ○ A group of connected pixels *C*_*p*_ are detected as seed points *SP* if size of *C*_*p*_ is larger than the user-defined size threshold *S*_*T*_

#### Biological seed modeling

**Figure 5:**
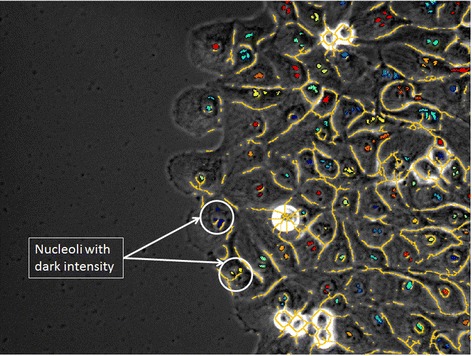
**Seed detection.** Nucleoli detection and clustering using the geodesic distance. Same color indicates nucleoli that belong to the same nucleus.

In order to increase the accuracy of detecting seed points, biological modeling of individual cells is incorporated into the seed detection algorithm. Nucleoli present in the nucleus area are usually dark and round when images are acquired using phase contrast modality as displayed in Figure 5. In contrast to the above technique, this method detects seed points at only one user-defined percentile threshold. The number of seed points remains constant between quantization levels. In our example the bottom 2% of the pixel intensity levels correspond to the nucleoli. The nucleoli are filtered by size as defined above using the user-defined size threshold *S*_*T*_. Additionally, they are also filtered by shape using a user-defined circularity threshold *C*_*T*_. The circularity is computed using the following formula: *C* = 4*π × area/(perimeter)*^*2*^. A valid seed point is a connected object with circularity above *C*_*T*_. Since multiple nucleoli can be present within one nucleus, a user-defined approximated diameter of the nucleus *D*_*N*_ is used to cluster multiple nucleoli together as part of the same nucleus. If the distance between respective nucleoli centroids is less than *D*_*N*_, then these nucleoli belong to the same cell. The distance between nucleoli can be computed as the Euclidian distance or the geodesic distance (user choice). The algorithm used to detect nucleoli as seed points is the following:

- Compute binary mask BW from user-defined percentile *t*: *BW = I > p(t)* or *BW = I < p(t)*,
- Apply pixel connectivity analysis to label the current mask,
- A group of connected pixels *C*_*p*_ are detected as seed points *SP* if size *and* circularity of *C*_*p*_ are larger than user-defined size threshold *S*_*T*_ and circularity threshold *C*_*T*_ respectively,
- Nucleoli with centroid distances smaller than *D*_*N*_ are assigned with the same label.

### Single cell boundary detection

Single cell boundary detection starts with the pixels identified as seed points. Unassigned pixels are then added at every percentile level. Pixels are assigned to the nearest seed point location by means of (1) the geodesic distance or (2) the Euclidian distance between the unassigned pixels and the boundary of the seed points.

**Figure 6:**
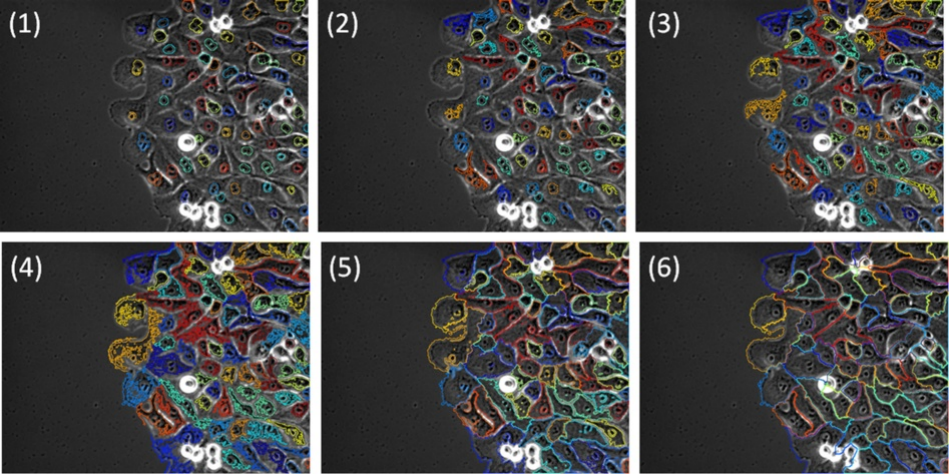
**Geodesic region growing steps.** Geodesic region growing for single cell edge detection starting from seed points and following the histogram percentile quantization of intensities in grayscale image and geodesic mask constraint. Images 1 to 6 are the masks generated from the 10th, 30th, 50th, 70th, 90th and 100th percentiles.

The geodesic pixel sorting technique improves single cell edge detection for boundary tracing close to a manually drawn one, as shown at some key steps in Figure 6, where the map chosen to perform the cuts is the grayscale image. The algorithm for border detection is as follows:

1. Begin from seed points,
2. Take the lowest (or highest) remaining bin of unmapped pixels and assign each to the seed point with the nearest boundary, where distance can be quantified by either Euclidean or geodesic distance,
3. Update boundary of seed points to reflect newly mapped pixels,
4. Repeat steps 2 and 3 until all pixels are mapped.

### Mitotic cell detection

**Figure 7:**
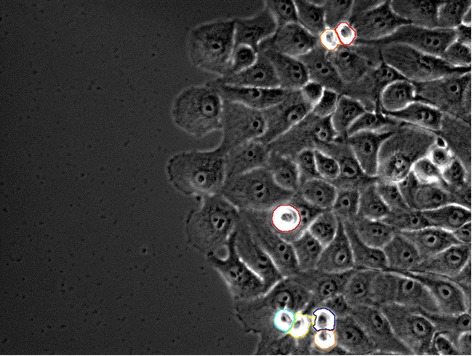
**Mitotic detection.** Mitotic Mask overlaid on top of the original phase image.

**Figure 8:**
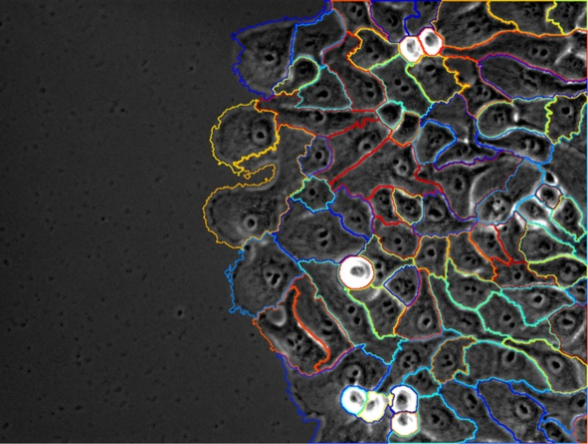
**Results.** Final segmentation result of the breast epithelial sheets.

For mitotic cell detection, we follow a model similar to the one presented in [33], where pixels with high intensities are detected by thresholding at a high intensity percentile value, and resulting clusters are tested for roundness. The mask generated by this technique is displayed in Figure 7. Thresholding for mitotic cells occurs at the 97th intensity percentile in that example. This mask is added to the last mask in Figure 6 and the final result is displayed in Figure 8. For more information about the value of the parameters chosen to perform this segmentation please refer to the Additional file 1. We performed a full factorial sensitivity analysis of these parameters in their full range presented in the Additional file 2.

## Results

In this section we compare segmentation performance of this new method with manually segmented datasets, as well as with other known techniques in this field.

### Reference datasets

In order to test the performance of the segmentation technique we used six datasets to create manual segmentation: (1) 10 phase images of bone cancer cells from Broad Institute [34] with a total of 2168 manually detected cells, (2) 10 Fluorescent images of E. coli cells from Duke University [35,36] with a total of 237 manually detected cells, (3) 10 Fluorescent images of yeast cells from Duke University [35,36] with a total of 153 manually detected cells, (4) 10 Fluorescent A10 rat cells from National Institute of Standard and Technology (NIST) with a total of 347 manually detected cells, (2) 10 phase images of NIH 3T3 cells from NIST with a total of 656 manually detected cells, and (1) 59 phase images of breast epithelial sheets from NIH with a total of 5722 manually detected cells.

A human expert manually segmented individual cells in each image of the reference datasets by drawing a boundary using a computer mouse and ImageJ software [37]. This reference data was inspected by a second expert to minimize human mistakes. It is available for download from https://isg.nist.gov/. Additional file 3 shows more details about the manual segmentation process.

### Measure of segmentation performance

The segmentation performance is measured using multiple metrics: (1) a cell count accuracy as used by Chowdhury et al. [38] that measures accuracy at a cellular level and (2) the Adjusted Rand Index as recommended by Bajcsy et al. [39] that measures accuracy at a pixel level.

The Cell Count Accuracy (CCA) metric is computed as follow:

$$ CCA=\frac{TP}{N+FP} $$

where *TP* is the True Positive count, the number of cells correctly detected by segmentation. *N* is the total number of cells manually detected. *FP* is the False Positive count, the number of cells detected by automated segmentation but does not exist in the manual one.

We report as complementary information: (1) Over-Segmentation, the number of cells that were split into multiple cells by the automated segmentation, (2) Under-Segmentation, The group of cells recognized as only one cell by the automated segmentation, and (3) The False Negative count, the number of cells that exist in the manual mask but are not detected in the automated one. This information is presented in the Additional file 4.

The Adjusted Rand Index (ARI) is used to evaluate the differences between the reference data and the automated segmentation results, following the procedure in [33]. The ARI measures similarities between two segmented images (labeled image1 and image2) at a pixel level, for images with multiple cells per image.

The adjusted rand index metric [40,41] is based upon counting the pairs of points on which two cell objects in both images agree or disagree. The ARI is bounded between 0 (no match) and 1 (best match) and is computed by the following formula:

$$ \begin{array}{c}\hfill ARI=\frac{{\displaystyle {\sum}_{ij}\left(\begin{array}{l}{n}_{ij}\hfill \\ {}2\hfill \end{array}\right)-\left[{\displaystyle {\sum}_i\left(\begin{array}{l}{a}_i\hfill \\ {}2\hfill \end{array}\right)}{\displaystyle {\sum}_j\left(\begin{array}{l}{b}_j\hfill \\ {}2\hfill \end{array}\right)}\right]/\left(\begin{array}{c}\hfill T\hfill \\ {}\hfill 2\hfill \end{array}\right)}}{\frac{1}{2}\left[{\displaystyle {\sum}_i\left(\begin{array}{l}{a}_i\hfill \\ {}2\hfill \end{array}\right)+{\displaystyle {\sum}_j\left(\begin{array}{l}{b}_j\hfill \\ {}2\hfill \end{array}\right)}}\right]-\left[{\displaystyle {\sum}_i\left(\begin{array}{l}{a}_i\hfill \\ {}2\hfill \end{array}\right){\displaystyle {\sum}_j\left(\begin{array}{l}{b}_j\hfill \\ {}2\hfill \end{array}\right)}}\right]/\left(\begin{array}{c}\hfill T\hfill \\ {}\hfill 2\hfill \end{array}\right)}\hfill \\ {}\hfill where\left(\begin{array}{c}\hfill a\hfill \\ {}\hfill b\hfill \end{array}\right)=\frac{a!\left(b-a\right)!}{b!}\hfill \end{array} $$

Let *C*1 denote the group of labeled cells in image1 and *C*2 the group of cells in image2. *T* is the total number of data points, *n*_*ij*_ is the number of overlapping pixels between cell *C*1_*i*_ in image1 and cell *C*2_*j*_ in image2, $$ \left(\begin{array}{l}{n}_{ij}\hfill \\ {}2\hfill \end{array}\right) $$ is a combination pair of data points, *a*_*i*_ and *b*_*j*_ are computed as follows:

$$ {a}_i={\displaystyle \sum_{j=1}^{k2}{n}_{ij}\kern0.3em } and\kern0.3em {b}_j={\displaystyle \sum_{i=1}^{k1}{n}_{ij}} $$

The background is being discarded from the ARI computation.

### Performance evaluation

We quantified the segmentation performance over the six reference datasets using the metrics mentioned above. We compared the performance of our new method against 5 other cell separation techniques available to us as open-source tools. In addition show the advantages of histogram quantization and the use of geodesic distance by including an additional technique, “FogBank-wopg,” without the use of these two techniques. There are a total of 7 total methods that are evaluated: (1) CellProfiler based on region growing [42], (2) CellTracer [35], (3) FogBank, (4) FogBank wopg, (5) Schnitzcells [43], (6) Frlbm using level sets [44], and (7) Marker-Controlled Watershed (MCW) [45]. The details of each pipeline can be found in the Additional file 1.

**Figure 9:**
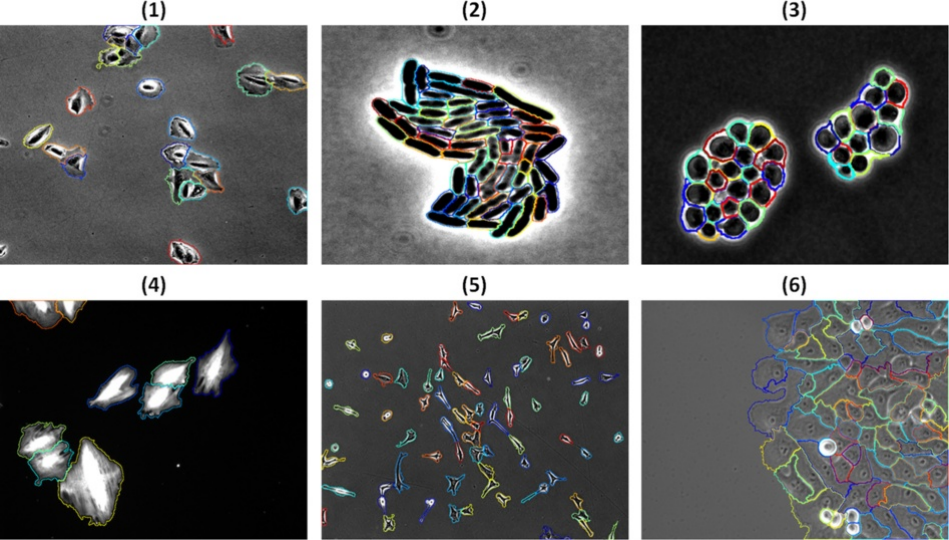
**Segmentation example.** Segmentation example of multiple image modalities and cell lines. The cell lines and image modalities used are: (1) phase images of bone cancer cells, (2) Fluorescent images of E. coli cells, (3) Fluorescent images of yeast cells, (4) Fluorescent A10 cells, (5) NIH 3T3 cells, and (6) breast epithelial sheet.

The geodesic distance concept for cell edge detection helped our segmentation obtain higher accuracies than the other methods, as it looks very similar to the manually drawn one in Figure 9. These high accuracies are attributed both to the quantization process, which eliminates the problem of over-segmentation, and to our method of tracking individual cell boundaries using geodesic distances to retain the shape of each cell within the image. A cell-by-cell comparison for all techniques, on example images from each reference dataset with the manual segmentation, are displayed in the Additional file 1.

**Figure 10:**
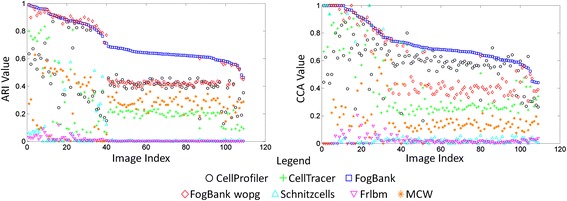
**Performance comparison.** Plots comparing the performance of all 7 methods over the entire reference datasets. The images are sorted with respect to the ARI and CCA values for method 3 (FogBank) respectively in each plot. This helps visualizing the differences between the other method and the new described method in this paper.

Figure 10 quantifies the differences between all 7 segmentation method results compared with manual segmentations over all 109 images of the reference datasets. The images on the x axis are sorted with respect to the ARI or CCA values from FogBank segmentation in each of the plots in Figure 10. The sorting makes it easier to highlight visually the difference between FogBank and other methods. One can notice that only a couple of points are above the squared blue line (representing FogBank results) in both the ARI and the CCA metrics. Tables 1 and 2 show the comparison results in a head-to-head matchup between methods. This table should be looked at per row for each method. The value in the element M(i,j) is the percent of reference images for which method i had a higher ARI or CCA than method j. These tables reveal the robustness of FogBank to segment single cells across image modalities and cell lines. With regards to comparing FogBank to FogBank-wopg, not only is FogBank more accurate than FogBank-wopg over 77% of the time as measured by both metrics, but also 10× faster in execution speed.

**Table 1:** **Method comparison using ARI**

**Methods**	**Cell profiler**	**Cell tracer**	**FogBank**	**FogBank wopg**	**Schnitzcells**	**Frlbm**	**MCW**
Cell Profiler	100	79.8	0.9	22.9	93.6	100	86.2
Cell Tracer	20.2	100	0	0	90.8	100	22.9
FogBank	99.1	100	100	77.1	100	100	100
FogBank wopg	77.1	100	22.9	100	100	100	100
Schnitzcells	6.4	9.2	0	0	100	43.1	9.2
Frlbm	0	0	0	0	56.9	100	6.4
MCW	13.8	77.1	0	0	90.8	93.6	100

Head-to-head comparison using the ARI metric between every pair of method.

The value in the M(i,j) element is the percent of images that method i had a higher ARI than method j.

**Table 2:** **Method comparison using CCA**

**Methods**	**Cell profiler**	**Cell tracer**	**FogBank**	**FogBank wopg**	**Schnitzcells**	**Frlbm**	**MCW**
Cell Profiler	100	67.9	8.3	55	90.8	100	100
Cell Tracer	32.1	100	1.8	4.6	91.7	100	90.8
FogBank	91.7	98.2	100	78	94.5	100	100
FogBank wopg	45	95.4	22	100	93.6	100	100
Schnitzcells	9.2	8.3	5.5	6.4	100	51.4	9.2
Frlbm	0	0	0	0	48.6	100	0.9
MCW	0	9.2	0	0	90.8	99.1	100

Head-to-head comparison using the CCA metric between every pair of method.

The value in the M(i,j) element is the percent of images that method i had a higher CCA than method j.

We applied the FogBank technique on 3 image modalities and 14 cell lines for a total of 876 images. The segmentation results are visually inspected and can be viewed and downloaded from the following webpage: https://isg.nist.gov/.

## Discussion

In order to efficiently extract biological information from images of confluent cells, highly accurate, automated methods for identifying and tracking individual cells in these images are needed. Particularly in heterogeneous cell population as they occur in tumor cell lines as well as in differentiating stem cell populations, the detailed analysis of individual cells over time will provide information of relevant biological properties. Cell lines used in biomedical research exhibit different morphology, and are additionally often used under conditions that alter their phenotype, for example change the cell shape from a polygonal to a spindle-like shape.

To address these issues, we developed a method that reliably and automatically identifies and tracks individual cells in cell sheets of vastly different origin such as bacteria, epithelial cells, and fibroblasts. Once cells are identified and tracked, additional analysis can be performed, e.g. for individual cells the migratory phenotype, protein expression levels, or changes in cell shape can be identified and used to characterize subpopulations of cells with distinct biological phenotypes.

In order to increase the accuracy of cell separation in images of confluent cells, we have directly addressed the problems with current watershed-like over-segmentation. By allowing watershed basins to grow in quantized increments instead of continuously across an intensity or gradient function, we reduce the noise associated with the continuous increment. In addition, we maintain the shape of individual cells during the process of growing the watersheds by using geodesic distance functions instead of a Euclidean distance function. If the algorithm can tell where cell boundaries lie, and use that information to form cell shapes, more realistic cell shapes will result.

The FogBank method does have some limitations: if cells are physically overlapping each other our method cannot separate them. In addition, although this method works very well on a number of different images modalities, such as phase contrast, bright field, and fluorescence microscopy images, it did not perform as well on Differential Interference Contrast (DIC) images. Nevertheless we feel that the accuracy we can achieve on other imaging modalities provides a contribution to the field of image analysis.

An open source Graphical User Interface (GUI) is created that allows the user to load a set of images from a specified location and visualize the segmentation on any image. It is created as a free standalone executable using MATLAB. This executable file (exe) requires the installation of the free MATLAB Compiler Runtime (MCR) that can be downloaded from the following link: http://www.mathworks.com/products/compiler/mcr/. All information and tools like the exe, the source code, and all datasets can be downloaded from the following link: https://isg.nist.gov/.

## Conclusion

We present a new technique called FogBank to separate individual cells in an image of a confluent sheet of cells or colonies. Our new method for separating single cells is highly accurate, on the order of 0.75 when compared with manually segmented cells. It can be applied on multiple image modalities and cell lines. We have compared our technique with other available techniques to show that the accuracy of our technique is higher than that of currently available algorithms. We demonstrated the use of this method on images of a wide variety of cell lines and image modalities. We provided an open-source user interface for the community to test this technique on an even wider range of applications.

## Additional files

Additional file 1:
**Pipeline and results of the reference dataset segmentation.** This Additional file describes in detail the pipelines used to segment single cells from all reference datasets as described in the main paper. The pipelines and the segmentation results described in this additional file come from 8 methods: (1) Fog Bank, (2) CellProfiler and (3) CellTracer. Additional file 2:
**Sensitivity analysis.** This Additional file describes the sensitivity analysis performed on the input parameters of the FogBank technique over a breast epithelial sheet image. Additional file 3:
**Detailed description of the manual reference dataset segmentation.** This Additional file describes in detail the creation of the reference datasets. We describe the step by step creation of the 6 manually segmented datasets by expert scientists. These masks are used to quantify the performance of the Fog Bank segmentation. Additional file 4:
**Segmentation performance evaluation results.** This Additional file is an excel spreadsheet that has all the detailed information about the segmentation performance for all methods and all datasets.
