# Supplementary material for: FogBank: a single cell segmentation across multiple cell lines and image modalities
Source: BMC Bioinformatics. 2014 Dec 30;15(1):431. doi: 10.1186/s12859-014-0431-x (PMC4301455; doi:10.1186/s12859-014-0431-x)
Supplement: Additional file 1: — Pipeline and results of the reference dataset segmentation. This Additional file describes in detail the pipelines used to segment single cells from all reference datasets as described in the main paper. The pipelines and the segmentation results described in this additional file come from 8 methods: (1) Fog Bank, (2) CellProfiler and (3) CellTracer. [file 12859_2014_431_MOESM1_ESM.docx]

FogBank: A Single Cell Segmentation across Multiple Cell Lines and Image Modalities

Joe Chalfoun^[[1]](#footnote-1)^, Mike Majurski^1^, Alden Dima^1^, Christina Stuelten^[[2]](#footnote-2)^, Adele Peskin^1^, and Mary Brady^1^

This Additional file describes in detail the pipelines used to segment single cells from all reference datasets as described in the main paper. The pipelines and the segmentation results described in this additional file come from 7 methods: (1) CellProfiler based on region growing, (2) CellTracer, (3) FogBank, (4) FogBank wopg, (5) Schnitzcells, (6) Frlbm using level sets, and (7) Marker-Controlled Watershed (MCW).

**Table of Contents**

[1 Fog Bank Pipeline 2](#_Toc403128644)

[2 CellProfiler Pipeline 7](#_Toc403128645)

[3 CellTracer Pipeline 18](#_Toc403128646)

[4 SchnitzCells Pipeline 23](#_Toc403128647)

[5 Frlbm using level sets Pipeline 24](#_Toc403128648)

[6 Marker-Controlled Watershed (MCW) Pipeline 26](#_Toc403128649)

[7 Segmentation Results per Dataset 27](#_Toc403128650)

[7.1 Bone Cancer Cells 27](#_Toc403128651)

[7.2 E.Coli Cells 27](#_Toc403128652)

[7.3 Yeast Cells 28](#_Toc403128653)

[7.4 A10 Cells 28](#_Toc403128654)

[7.5 3T3 Cells 29](#_Toc403128655)

[7.6 Breast Epithelial Cells 29](#_Toc403128656)

# Fog Bank Pipeline

After loading the images, we begin the process of separating single cells by locating the foreground pixels using the Empirical Gradient Threshold (EGT) technique [1] with the set of parameters as displayed in Figure 1.


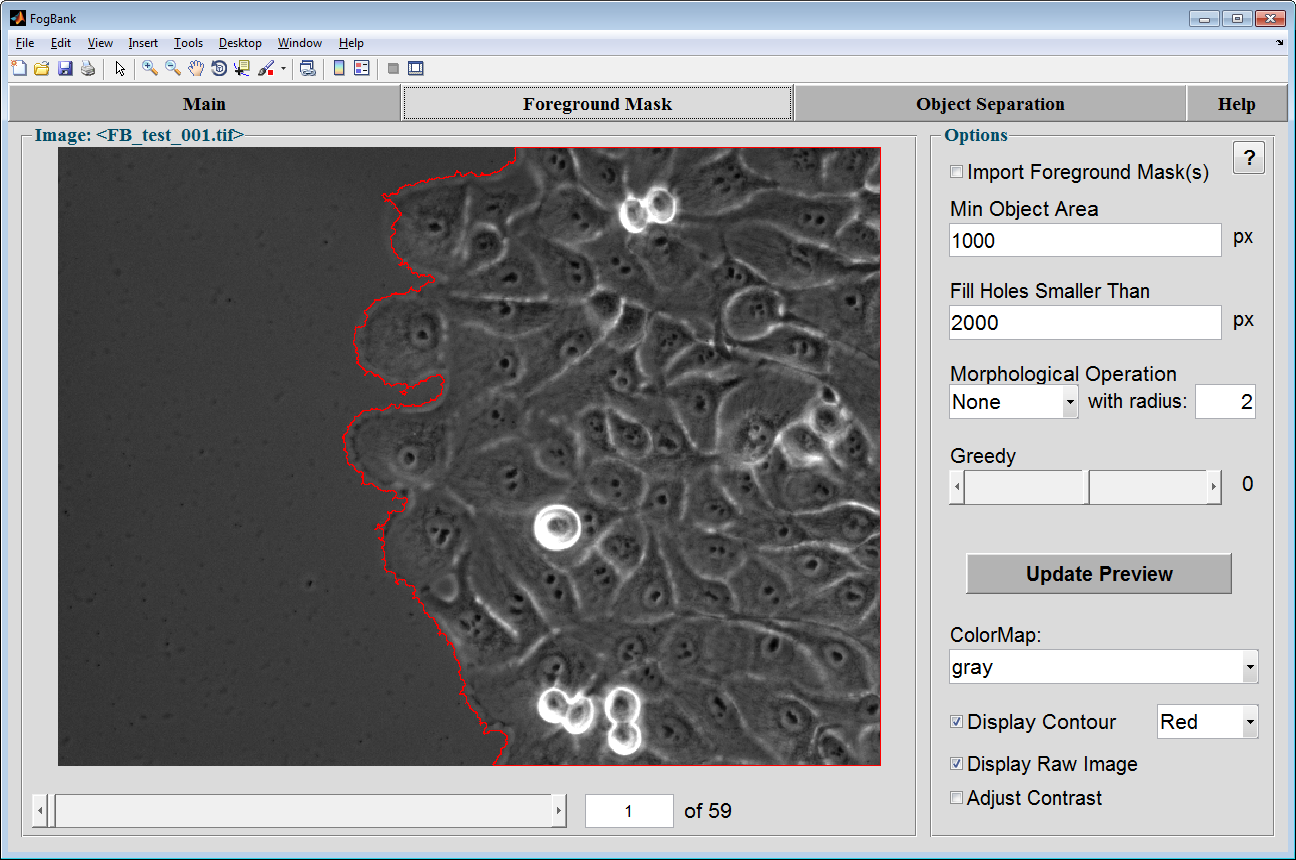


Figure 1: Foreground background segmentation performed using EGT

The geodesic distance concept helps to detect single cell boundaries similar to a manually drawn ones. Figure 2 shows the geodesic mask overlaid on the original phase image where the red pixels are the boundaries that cannot be traversed. Boundaries are defined through a user input percentile threshold, where the boundaries are considered to have high pixel intensities. In our case, the boundaries are composed of pixels with intensities higher than the 90^th^ percentile intensity. This initial border mask between the cells is cleaned up using morphological processing. Dilation with a disk of 1 to expand the borders, bridge to close single pixel gaps, thin to preserve only the borders as a single pixel line, and diag to make the borders 8 connected. At this point any holes in the mask were broken to prevent trapping a seed within a ring in the border mask. The result is a binary mask image where true pixels are borders between cells. This mask will also be used to cluster the seed points generated by the seed detection tab.


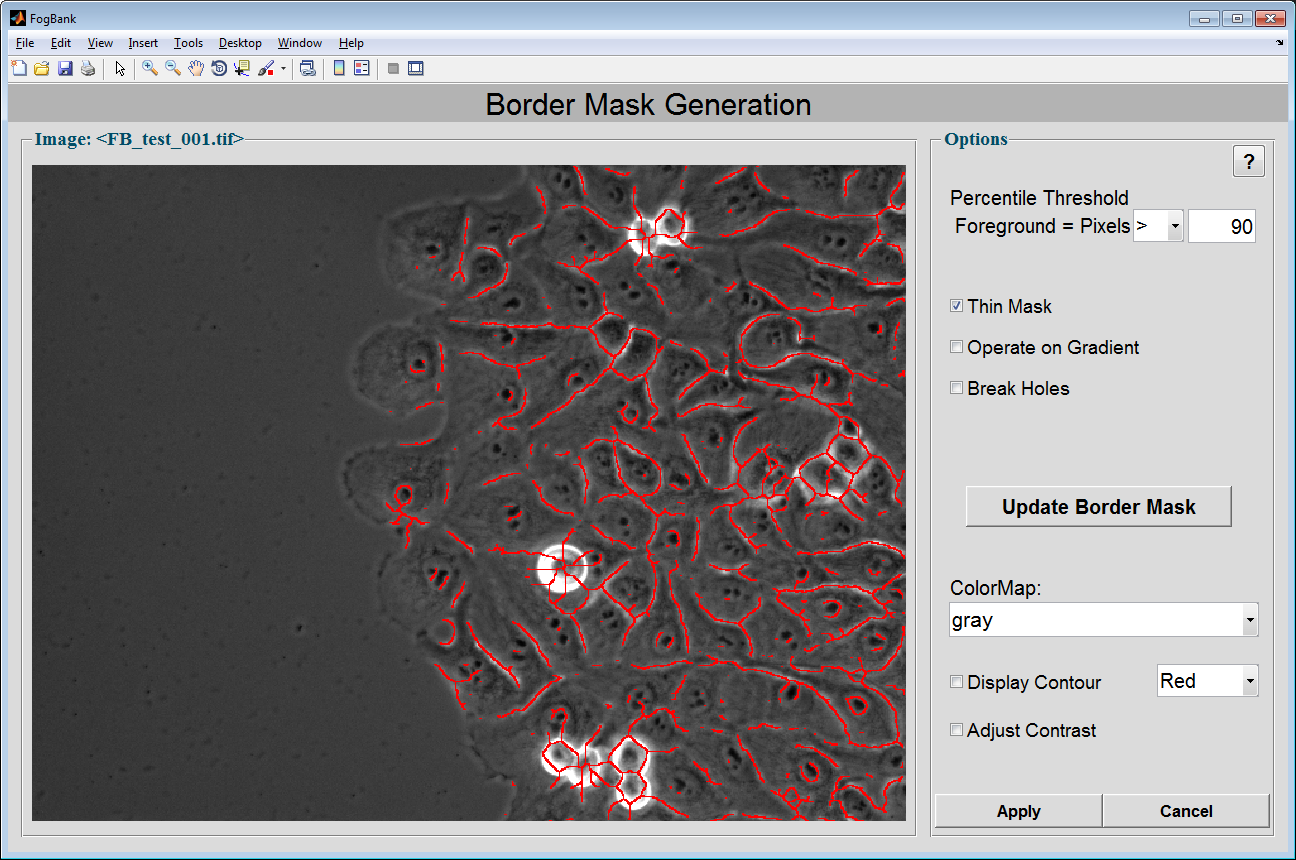


Figure 2: Finalized Border Mask

The detection of seed points determines whether an image is over or under-segmented. In order to increase the accuracy of detecting seed points, the user has the choice of segmenting them with a double thresholding technique and morphological operation or simply uploading the seed masks in the GUI. In the case of breast epithelial cells, the nucleoli present in the nucleus area are usually dark and round when images are acquired using phase contrast modality. The nucleoli are detected as being the darkest 1.8% of all pixel intensities and then filtered by size with minimum size of 7 pixels. Since multiple nucleoli can be present within one nucleus, a geodesic distance of 20 is used to cluster multiple nucleoli together as part of the same nucleus. Any set of seed objects whose centroid geodesic distances are less than 20 are grouped into a single cell object and are all given the same label.


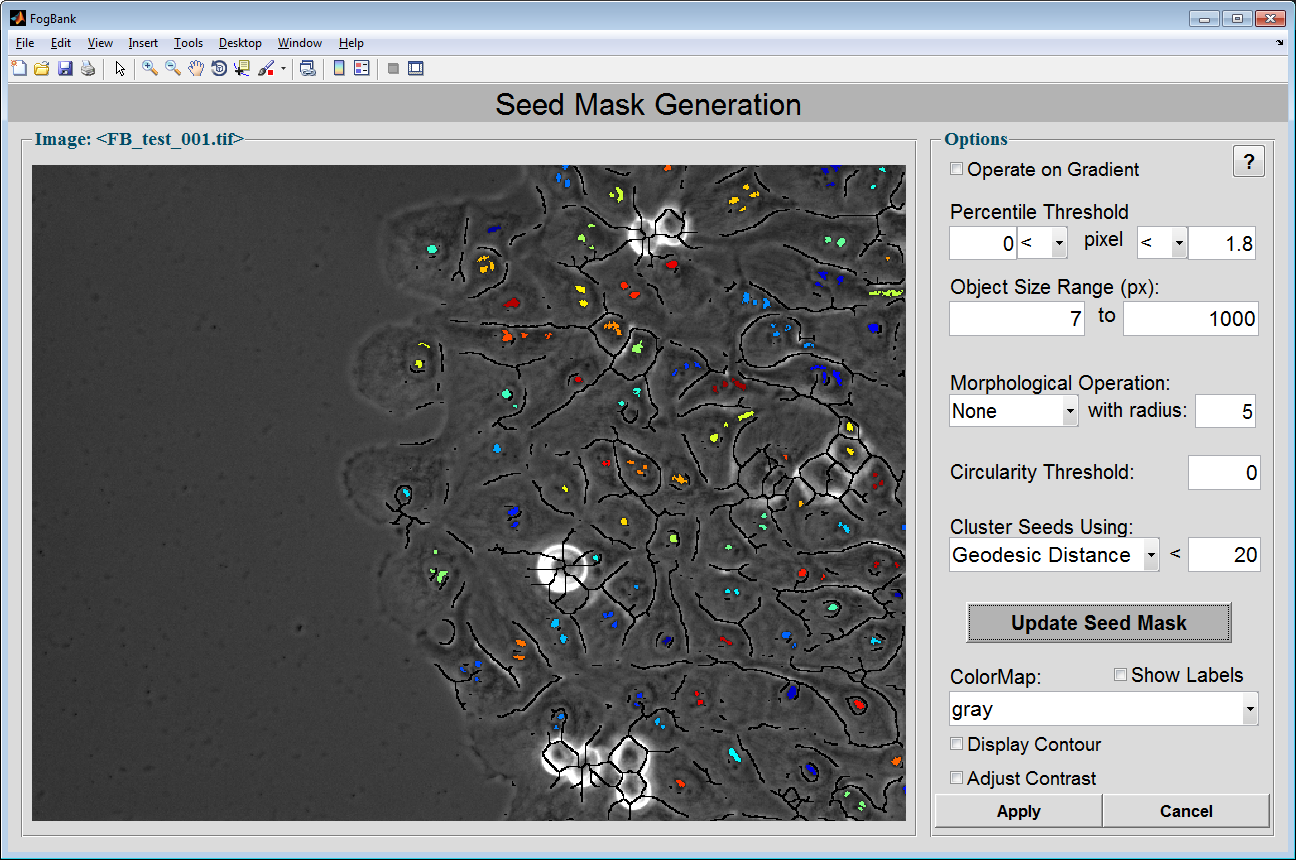


Figure 3: Initial Seed locations segmented respecting the border mask

In the breast epithelial cell sheets, most dark nucleoli have a brighter halo surrounding them in the raw phase contrast images (Figure 4). Fogbank works by incrementally adding pixel mass to a known object by iterating over a percentile threshold. This iteration starts at the lowest percentile threshold and works its way higher. As more and more pixels meet this threshold requirement they are added to the nearest connected body (seed in this case). This results in the dark pixels being grabbed first, then brighter pixels later. The effect of the halo on this algorithm is that it traps the object until the threshold passed the level of the halo. To get around this we dilate the seeds, taking into consideration the border mask. Figure 5 shows the final seed mask.


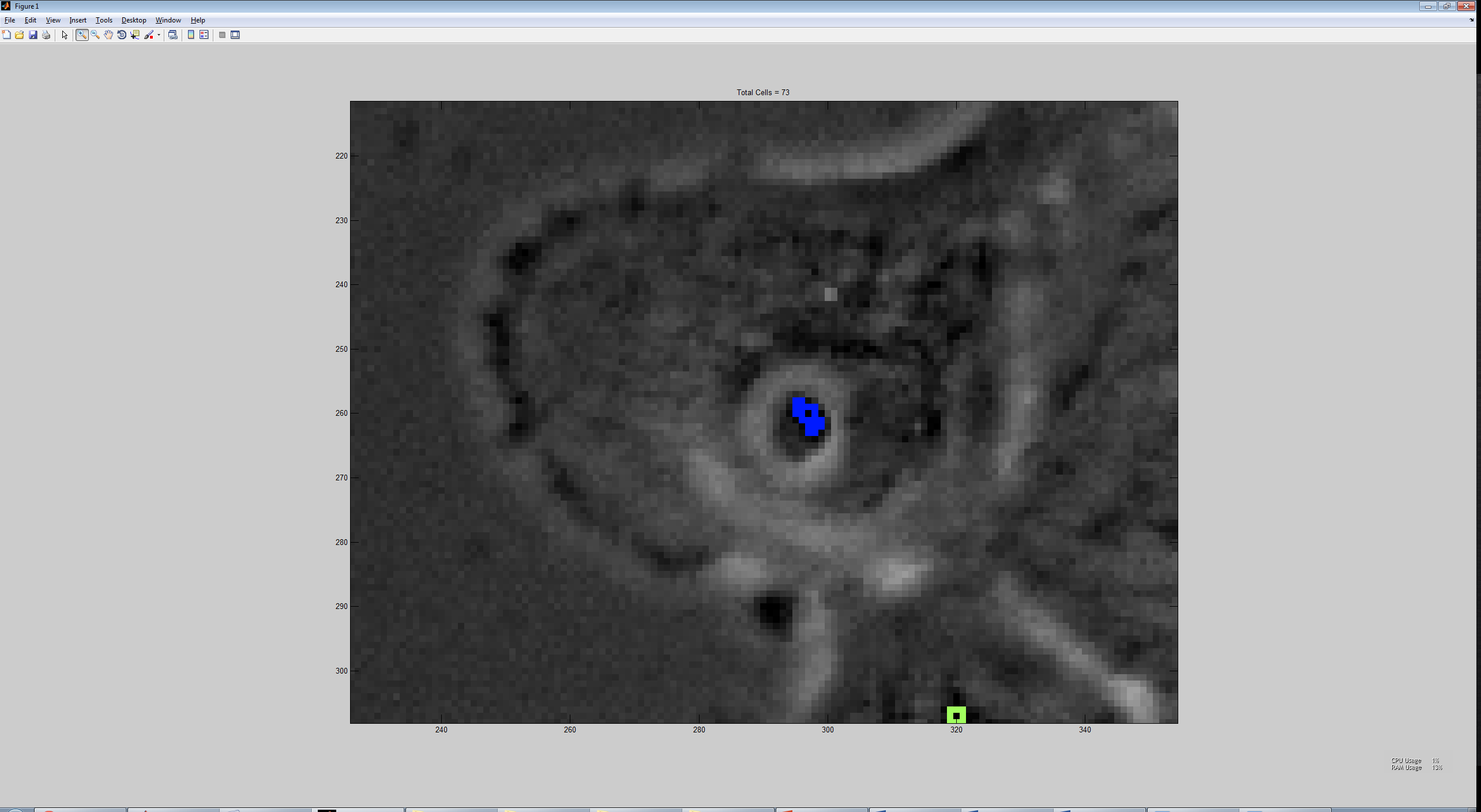

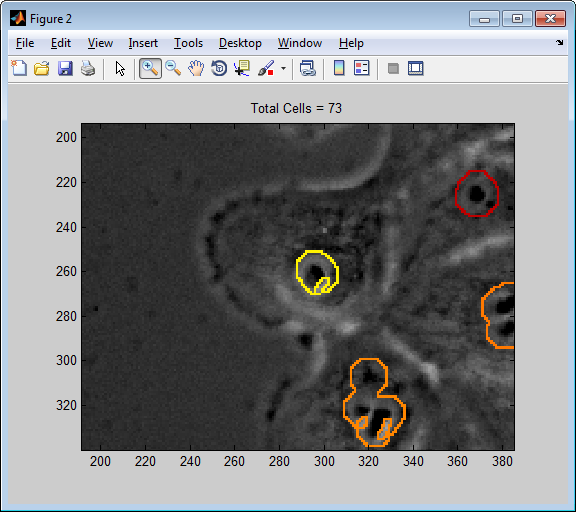


Figure 4: Initially detected seed (left); seed dilated to overcome seed halo (right)


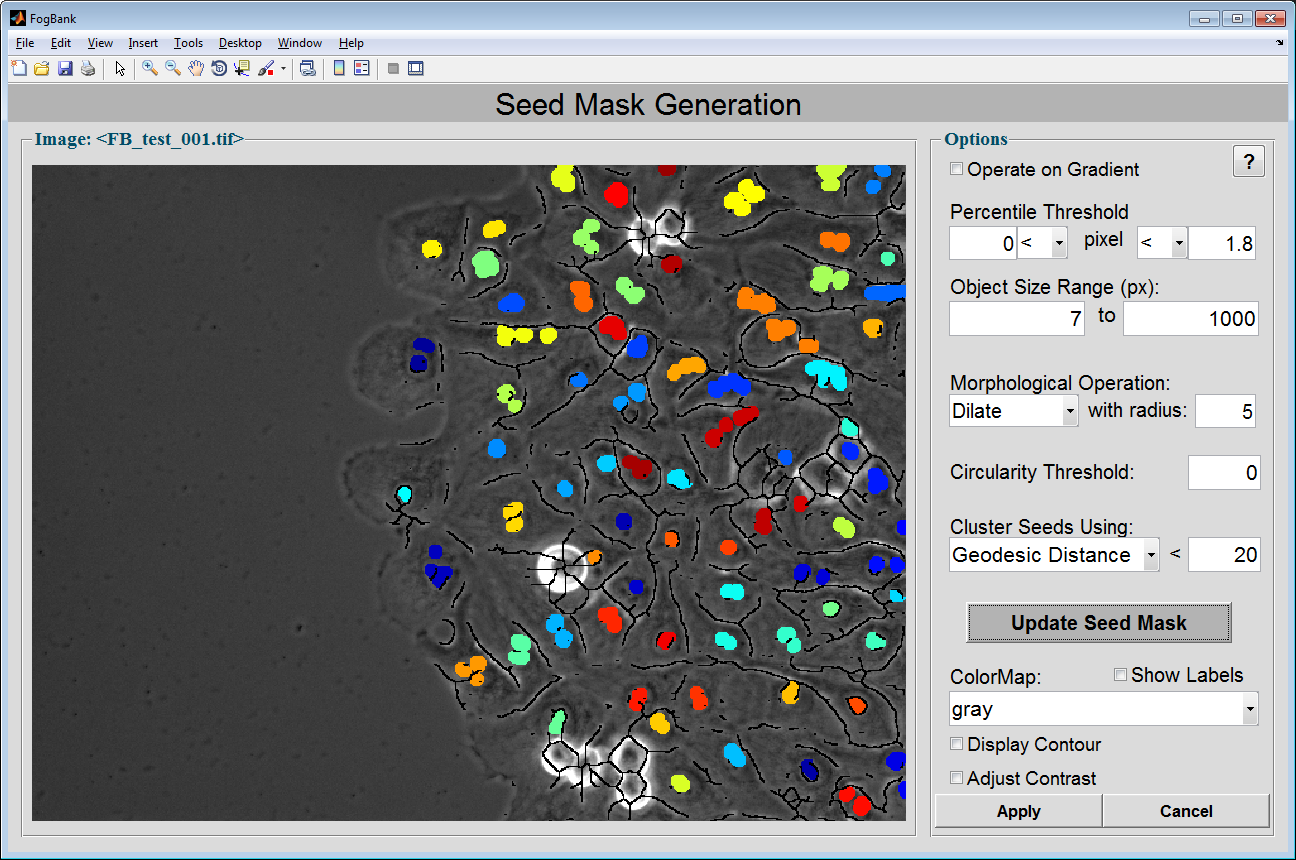


Figure 5: Seeds dilated to expand past the halo surrounding the dark nucleoli

At this point the seeds are ready to form the basis of the single cell segmentation. The FogBank algorithm is run iterating from the dark pixels to the bright pixels with a minimum object size of 300 pixels. The result is an approximation of single cell segmentation where the mitotic cells are missing.

The mitotic cells are extracted by thresholding the foreground pixels at the 97^th^ percentile within a minimum object size of 300. The mitotic mask is then cleaned up using morphological open with a disk of radius 8 to remove the thin objects, but leave the large mass, mostly circular objects. The touching mitotic cells are then cut apart using fogbank with a minimum object size of 300 pixels and a minimum peak size of 120 pixels as displayed in Figure 6.


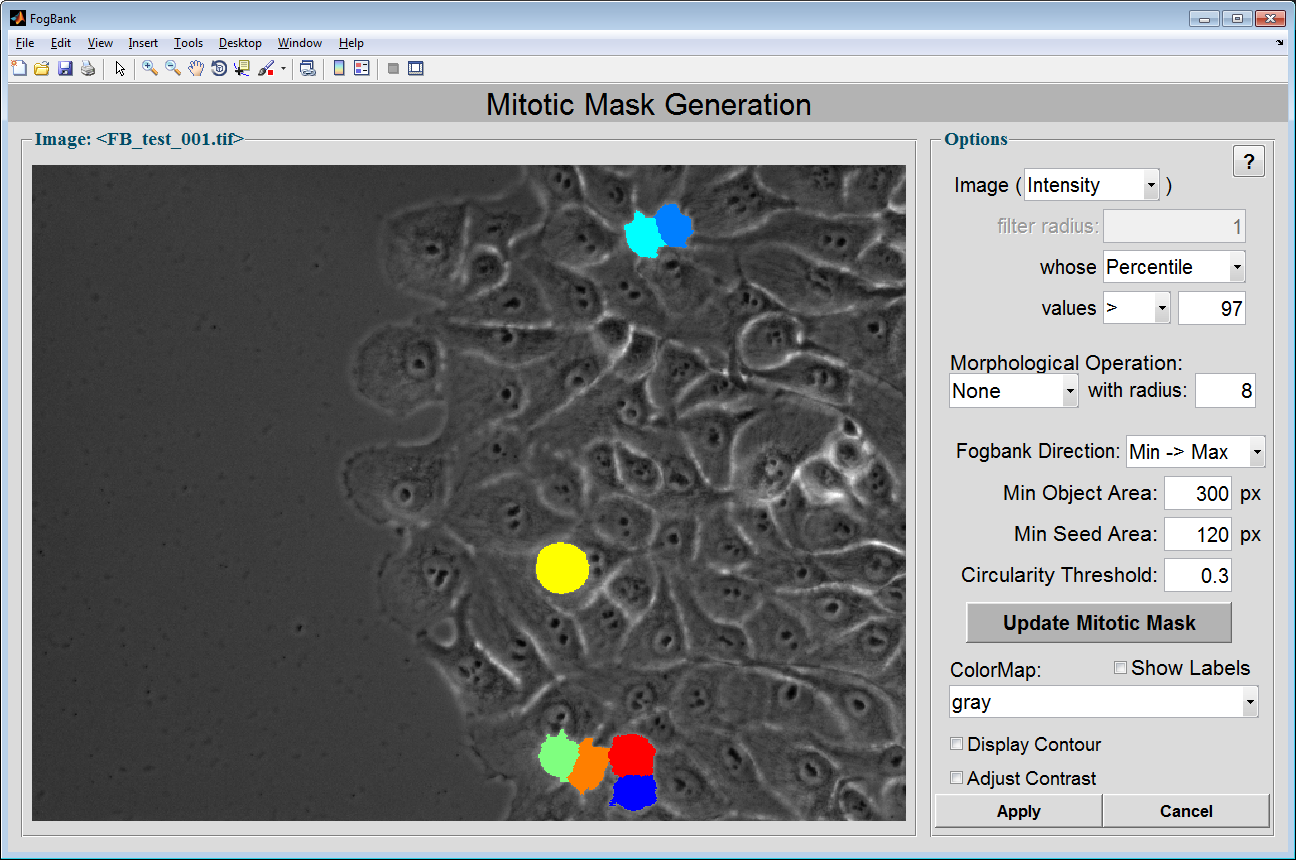


Figure 6: Mitotic cells segmented from the sheet

The mitotic cells are then added to the segmentation results to produce the final segmentation shown in Figure 7.


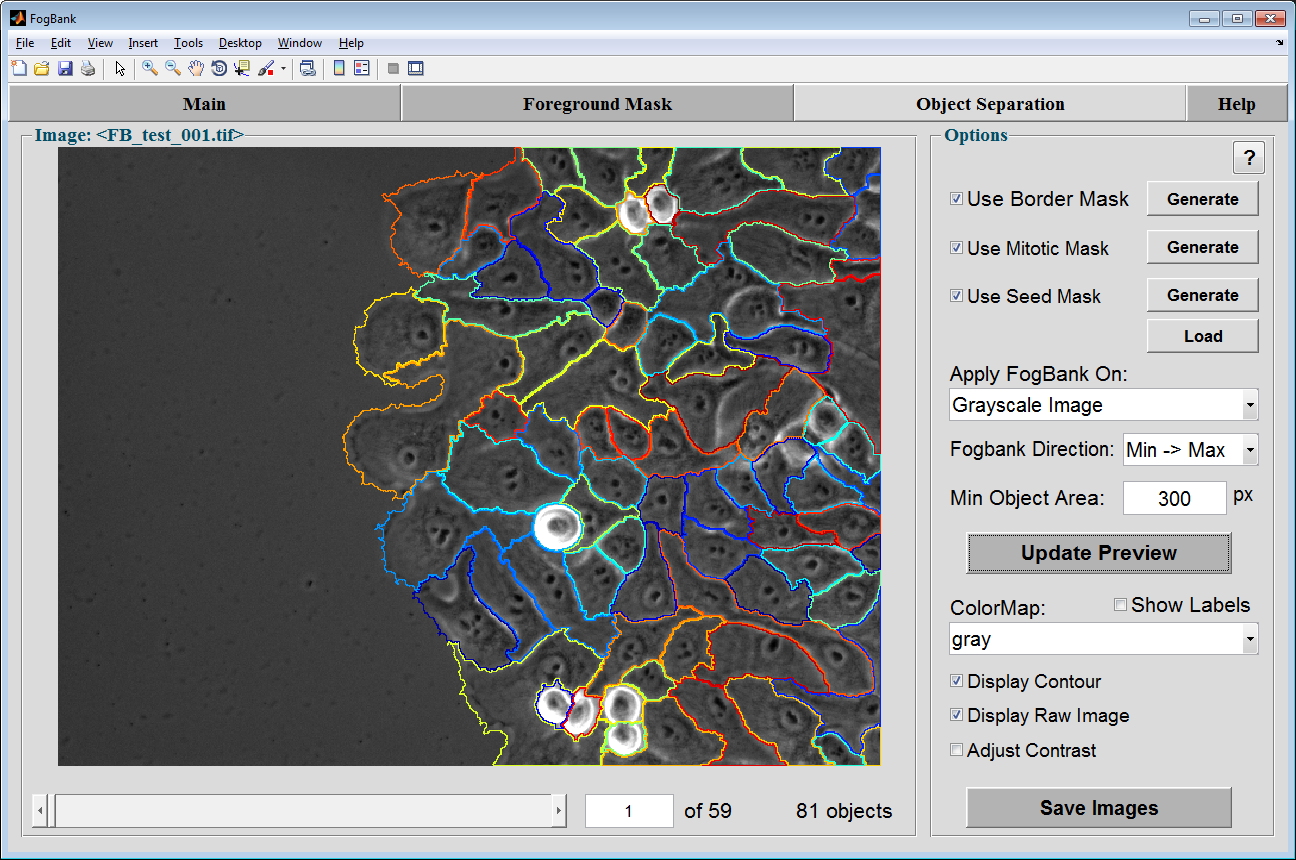


Figure 7: Final segmentation of the cell sheet


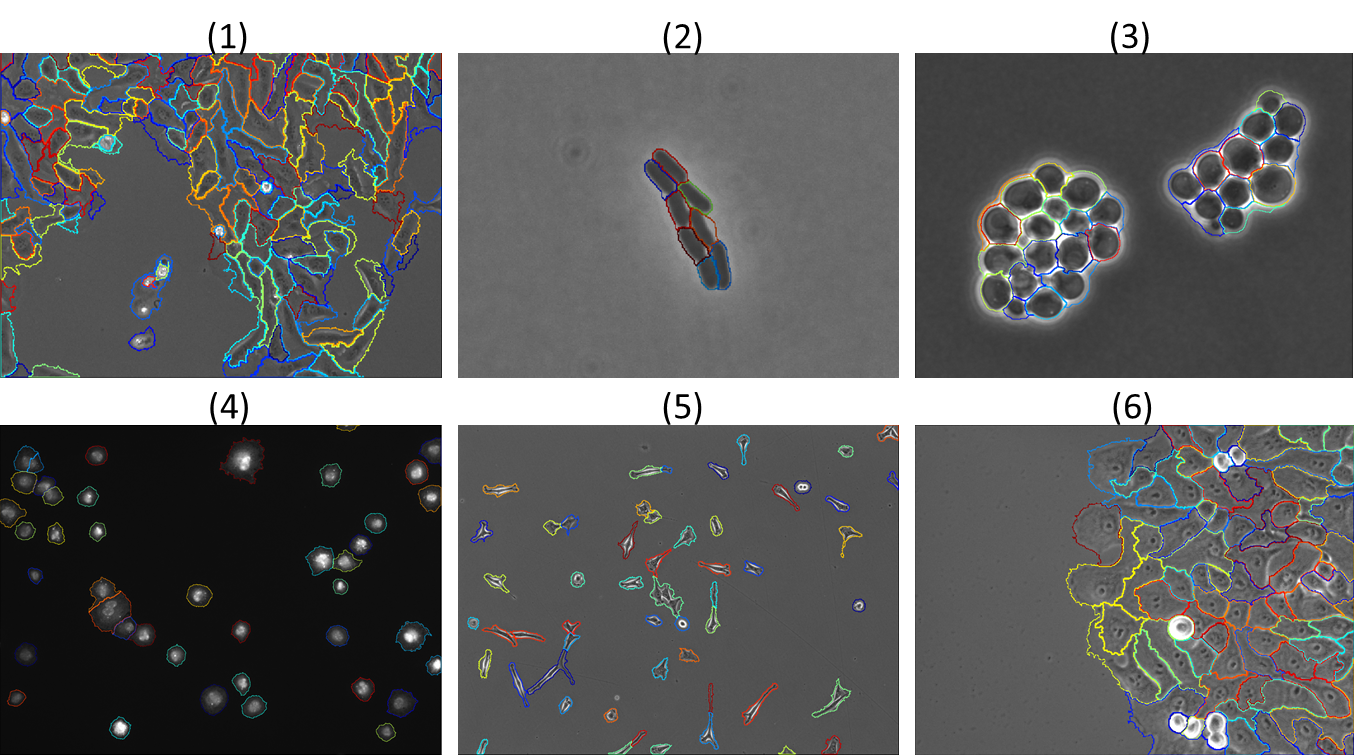


Figure 8: Example Fogbank Segmentation Results. (1) Bone Cancer Cells, (2) E.Coli Cells, (3) Yeast Cells, (4) A10 Cells, (5) 3T3 Cells (6) Breast Epithelial Cells

# Fogbank wopg Pipeline

This version of the Fogbank segmentation is performed with the exact same pipeline as the regular Fogbank with the following changes: 1) no border mask is generated, 2) Euclidean distance is used to assign pixels to the nearest object when dropping the fog level as opposed to geodesic distance, 3) percentile binning is not used for aggregating pixels together into bins when dropping the fog levels.


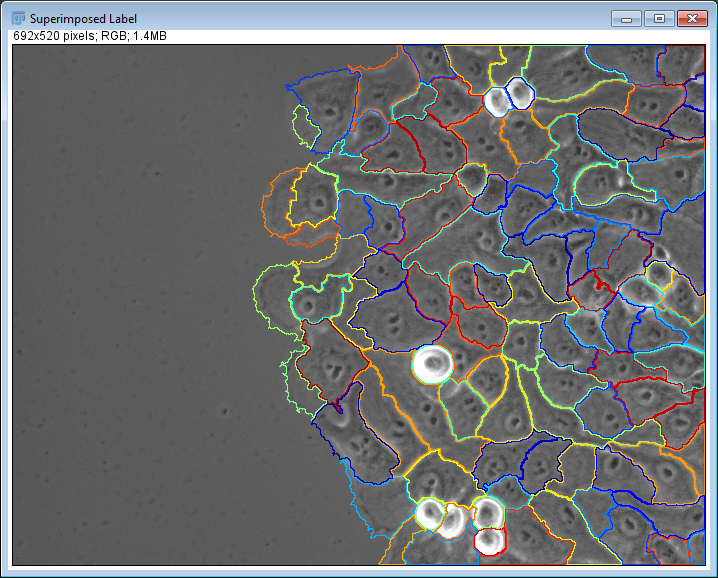


Figure 9: Segmentation Result when using Fogbank without geodesic distance.

# CellProfiler Pipeline

The first step in performing single cell segmentation is to determine which pixels are foreground (cell material) and which pixels are background. The method applied here is a gradient based technique to find the edge of the cell sheet and then fill in the foreground holes since it is known a priori there are no holes in the cell sheet. CellProfiler can compute the gradient of the image using the EnhanceEdges module, which applies the Sobel Operator to the input images.


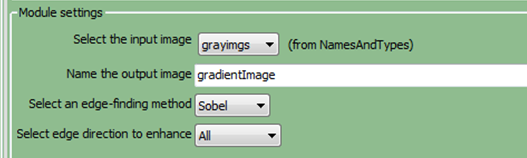


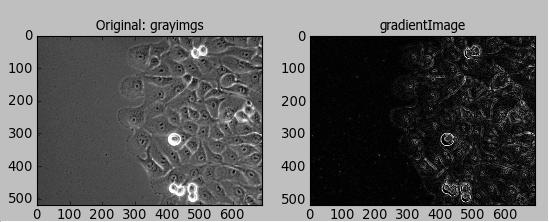


Figure 10: CellProfiler EnhanceEdges Module

The ApplyThreshold module is then used to threshold the gradient image into a binary mask using the RidlerCalvard thresholding method included with CellProfiler.


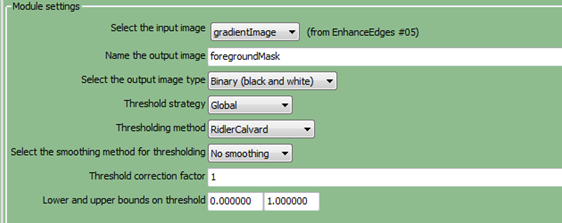


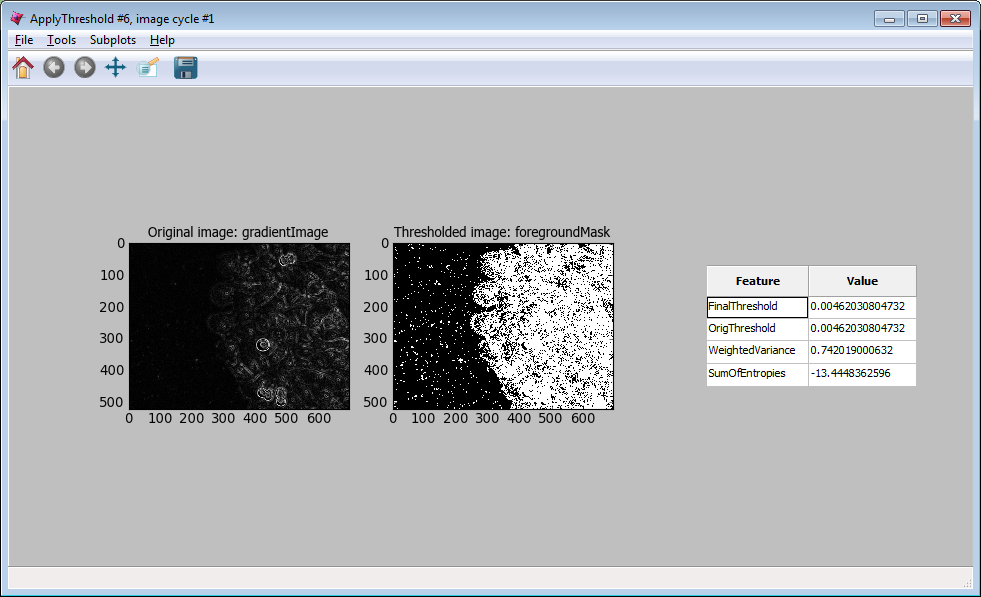


Figure 11: CellProfiler ApplyThreshold Module

The foreground mask image is hole filled to remove any holes smaller than 5000 pixels. This hole filling is accomplished using the Morph module that performs morphological operations on a binary image. The hold size threshold of 5000 was selected manually, and reflects the a priori knowledge that there are no small holes in the cell sheet. This step could easily be replaced with a generic hole filling where a hole is defined as a background region that is not 4 connected to the edge of the image through only background pixels.


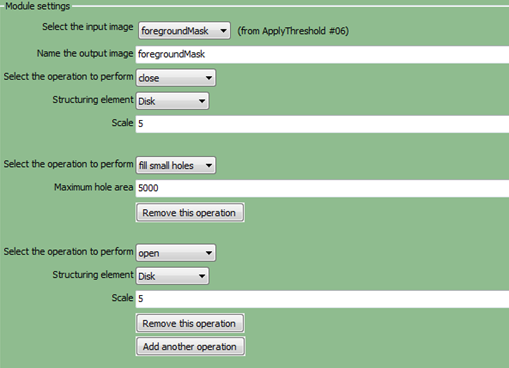


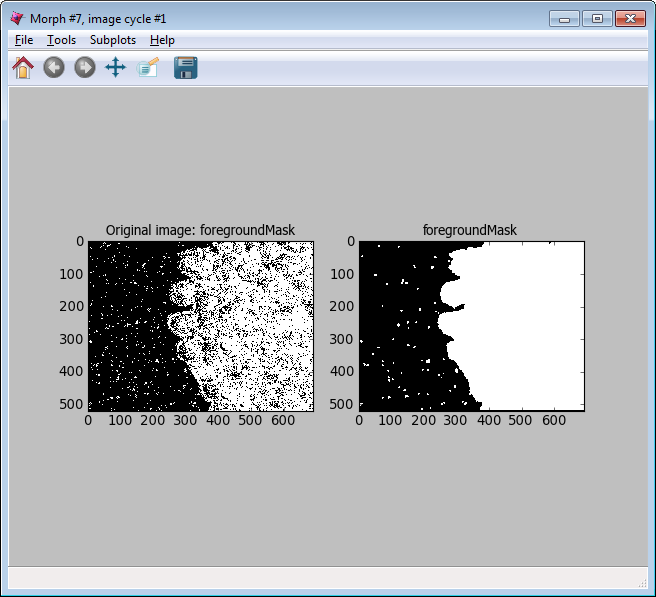


Figure 12: CellProfiler Morph Module to setup to perform hole filling

Small objects in the foreground mask are removed using the Morph module to prevent small objects, which are most likely noise or segmentation error, from influencing the results. The minimum object size specified here is 1000 pixels, a value chosen manually based on the rough size of a small single cell, in case a single cell gets separated from the sheet.


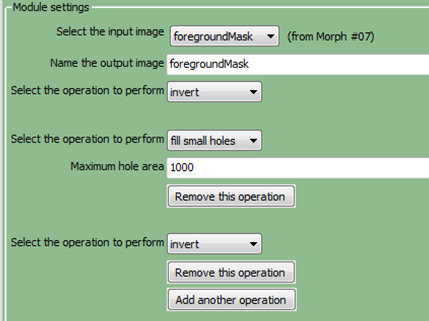


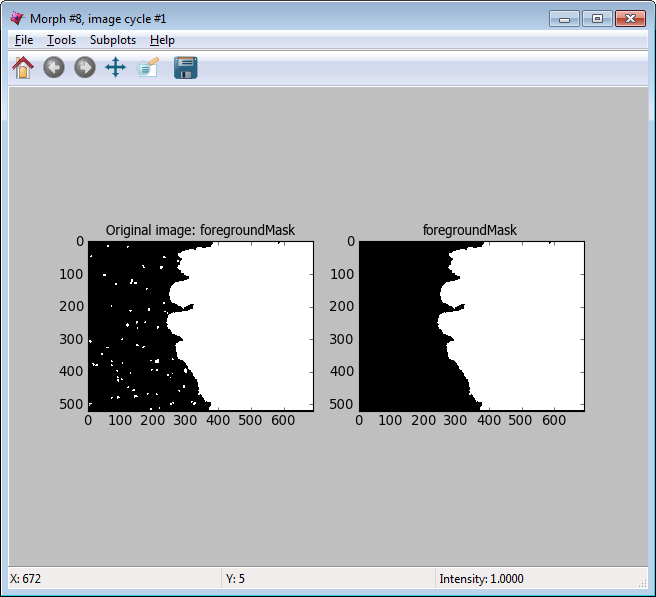


Figure 13: CellProfiler Morph Module setup to remove objects smaller than a minimum size

At this point the foreground has been extracted from the input phase contrast image, so the non-foreground pixels in the input images are suppressed using the CellProfiler module MaskImage. Any pixel in the input grayscale image that is not true in the binary segmented foreground mask image is labeled as invalid.


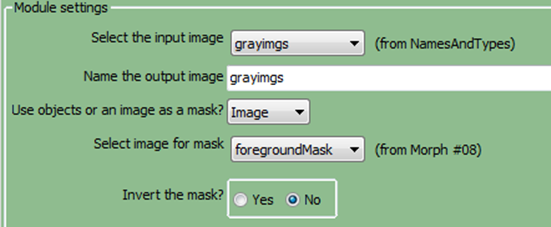


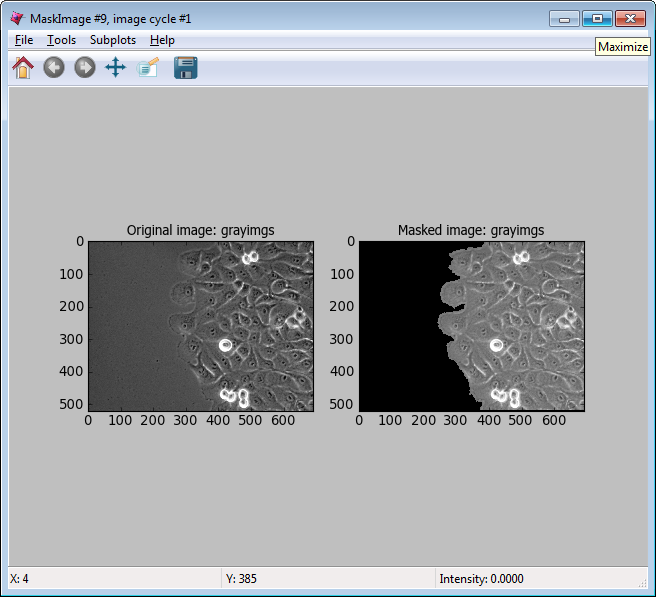


Figure 14: CellProfiler MaskImage Module to remove non foreground pixels from the input image

The module RescaleIntensity is then used to expand the masked images pixel values to use the full intensity range available now that the non-foreground pixels have been removed.


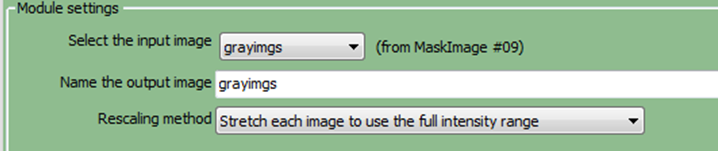


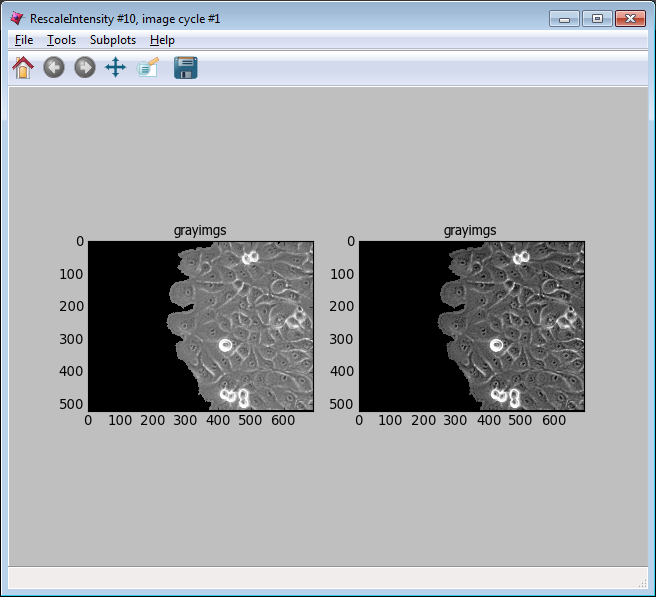


Figure 15: CellProfiler RescaleIntensity Module to expand the masked image to use its full intensity range

The foreground pixels are then inverted to convert the dark cell nuclei into bright peaks. This is done to allow CellProfiler’s Identify Primary Objects module to extract out these cell nuclei as seeds for finding cells within the sheet. Identify Primary Objects can threshold a grayscale image, however it can only consider objects as brighter than the threshold as foreground, and because we are trying to extract the dark nuclei of the cells, the images need to be inverted.


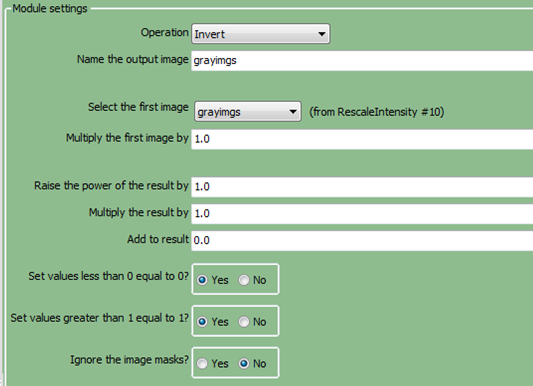


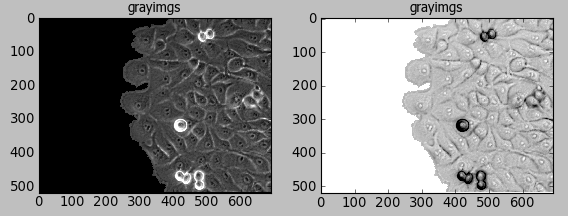


Figure 16: CellProfiler ImageMath Module to invert the image intensities.

The foreground-background mask is then reapplied to suppress the non-foreground pixels in the inverted image. Inverting the image re-validated these pixels and gave them a value equal to the maximum value in the image.


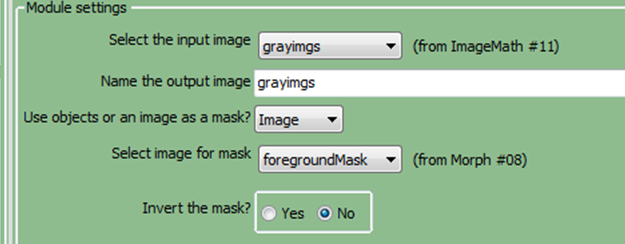


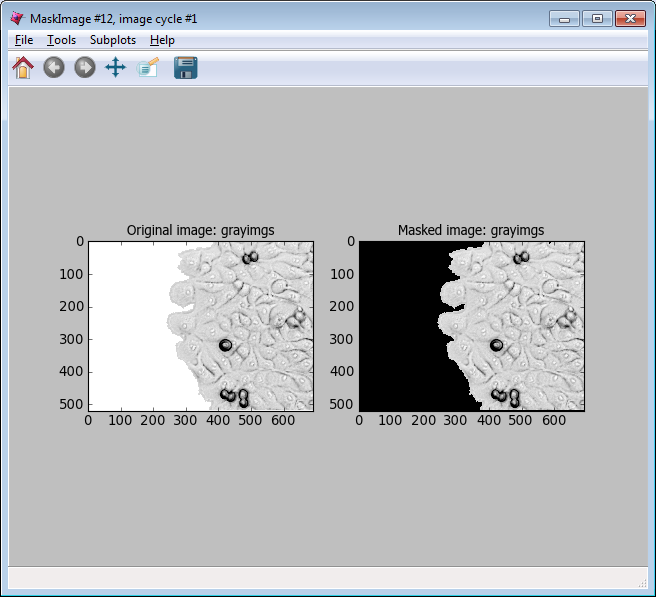


Figure 17: CellProfiler MaskImage Module to remove non foreground pixels from the input image

The nuclei are extracted using CellProfiler’s identify primary objects module, with the parameters manually set to extract objects above an intensity threshold that are roughly the size the nuclei are known to be.


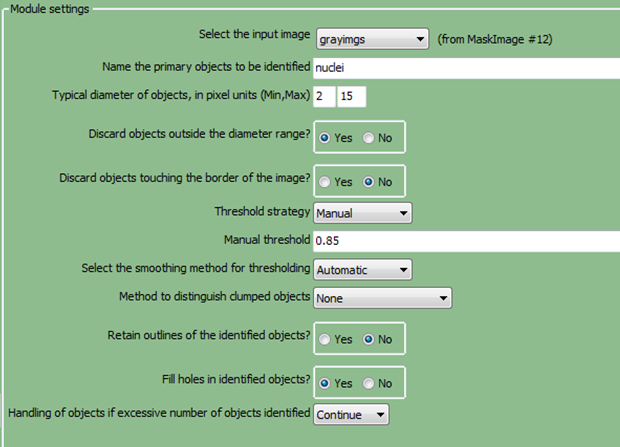


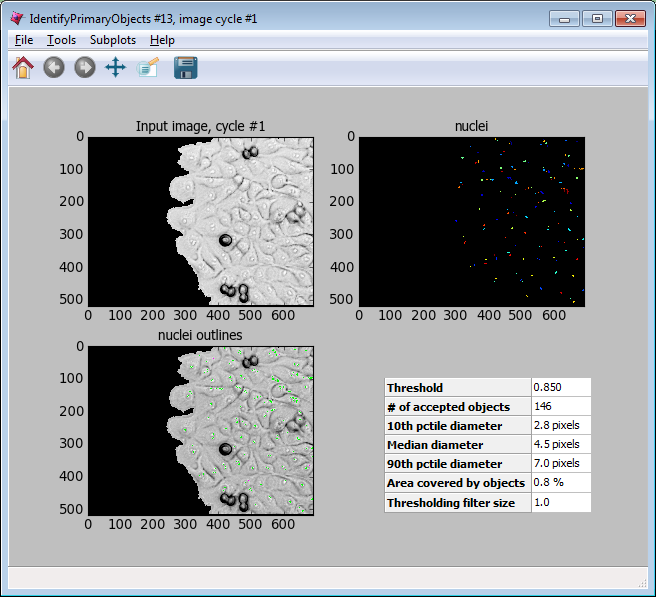


Figure 18: CellProfiler IdentifyPrimaryObjects Module to find cell nuclei

The primary objects that were detected are dilated to expand them past the bright halo that is known to exist surrounding nuclei objects. The goal of expanding the cell nuclei is to prepare for the find secondary objects CellProfiler module, preventing the bright halos surrounding the dark nuclei (dark halos and bright nuclei once the image is inverted) from interfering with the pixel assignments.


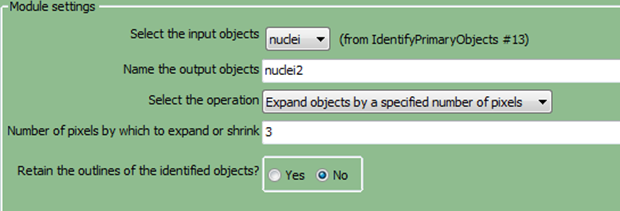


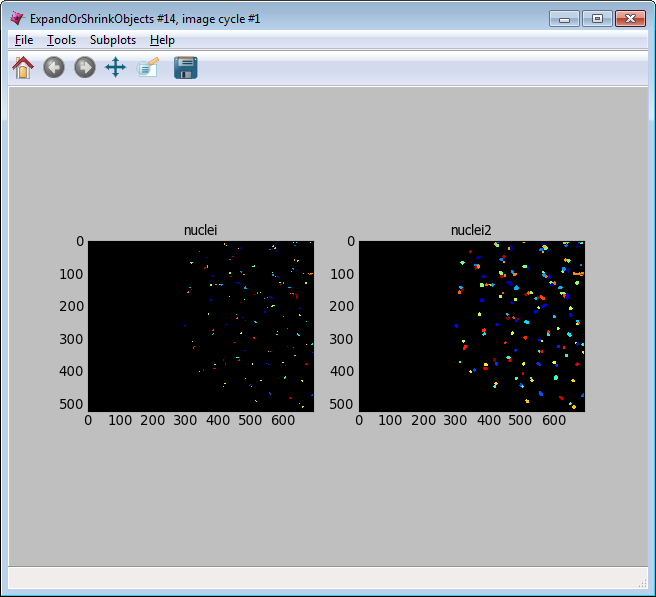


Figure 19: CellProfiler ExpandOrShrinkObjects Module to dilate the detected nuclei

Cell nuclei are clustered using a manually set distance to account for the fact that a single cell can have multiple nuclei objects within it. Having multiple primary objects detected for a single cell would cause over-segmentation errors, so clustering the nuclei is an attempt to solve this problem.


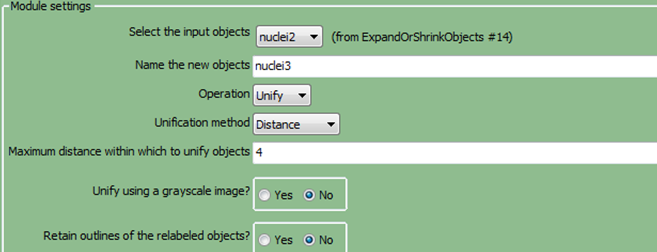


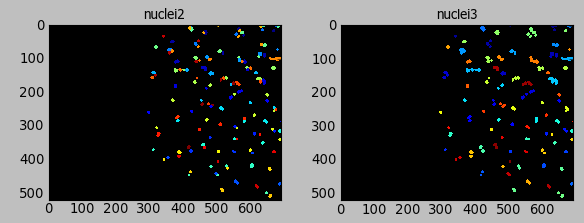


Figure 20: CellProfiler ReassignObjectNumbers Module to relabel the nuclei, clustering them into groups

CellProfiler’s identify secondary object module is then used to assign pixels to the proper primary objects (nuclei) to fill in the rest of the cell body.


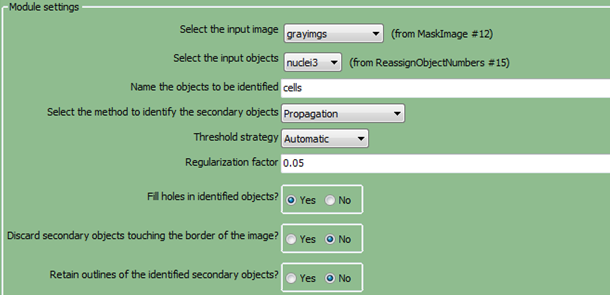


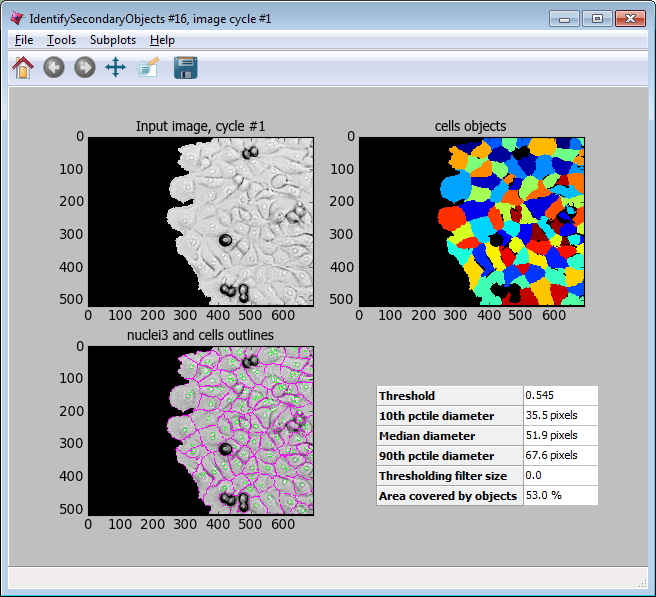


Figure 21: CellProfiler IdentifySecondaryObjects Module to assign the remainder of the foreground pixels to a primary object

The cells that have been segmented into CellProfiler objects are converted into a labeled mask image to be saved to disk.


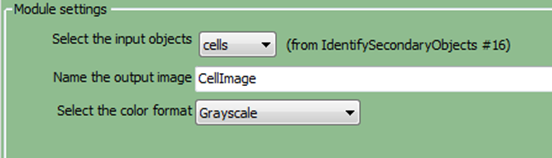


Figure 22: CellProfiler ConvertObjectsToImage Module to create a segmented mask image

The segmented images are saved off to disk as labeled masks to allow comparison to other segmentation techniques.


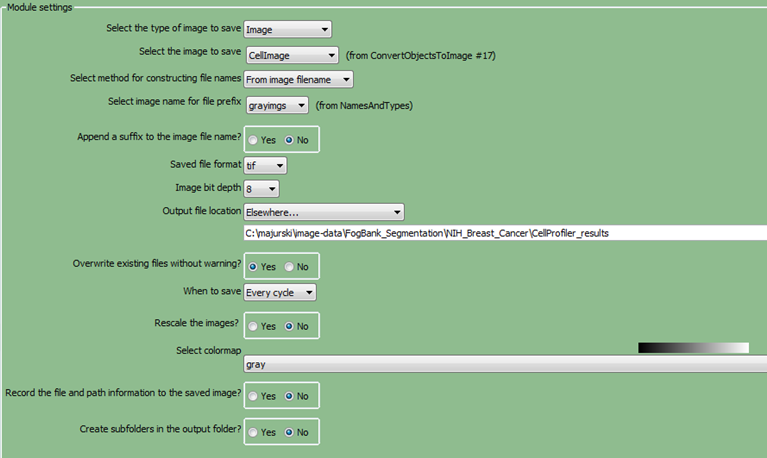


Figure 23: CellProfiler SaveImage Module to save labeled segmented Images to disk


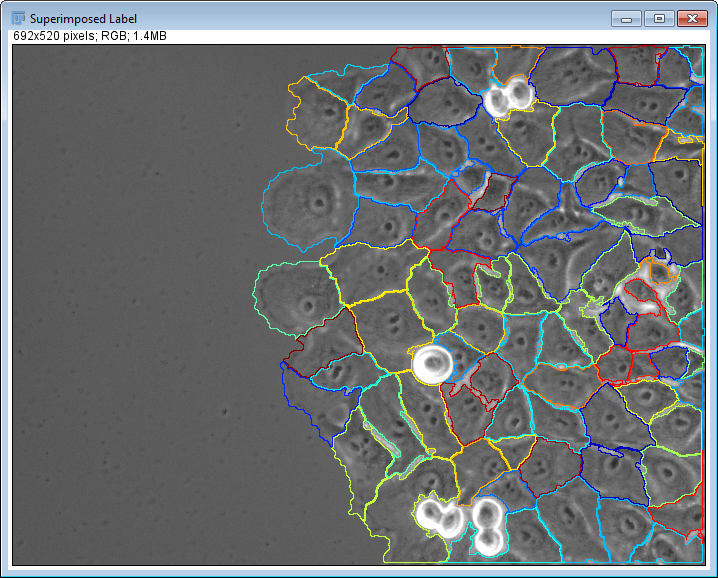

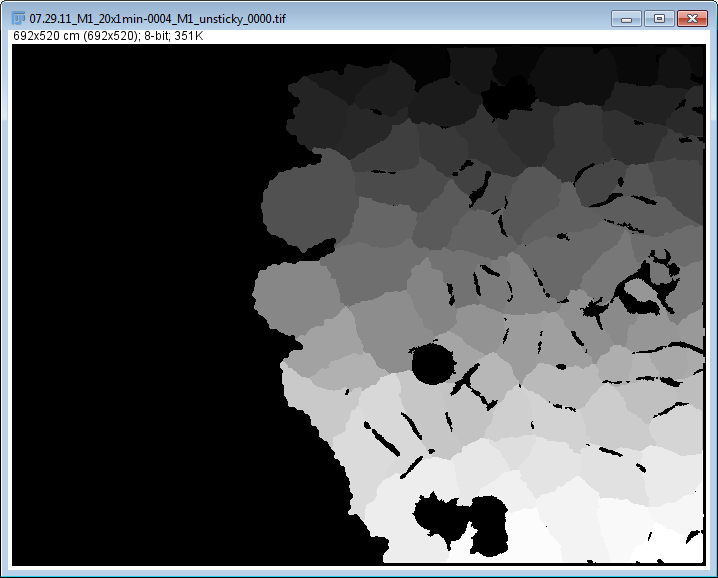


Figure 24: Resulting segmentation mask (left); Superimposed segmentation (right)


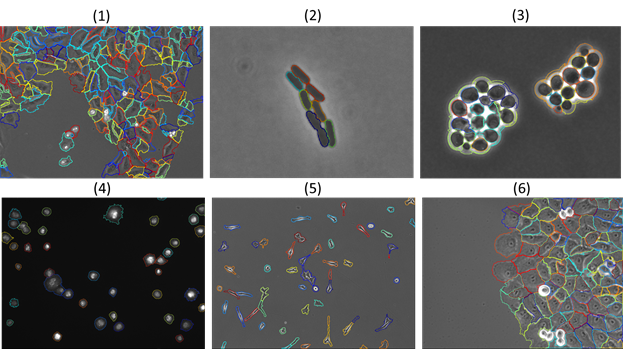


Figure 25: Example CellProfiler Segmentation Results. (1) Bone Cancer Cells, (2) E.Coli Cells, (3) Yeast Cells, (4) A10 Cells, (5) 3T3 Cells (6) Breast Epithelial Cells

# CellTracer Pipeline

CellTracer is a collection of Matlab segmentation and tracking algorithms packaged with a GUI front end that can be used to automate cell segmentation [2]. CellTracer is available free of charge from <http://www.stat.duke.edu/research/software/west/celltracer/>.

The test image sequence was loaded into CellTracer by opening a new project, navigating to the directory holding the evaluation images, and selecting the set of test images.

Figure 26: input phase contrast images loaded into a new CellTracer project

The first step in performing single cell segmentation is to determine which pixels are foreground (cell material) and which pixels are background. That was accomplished using CellTracer’s segmentation algorithm called “Background Range Filtering” using the following parameters:

Max Cell Half Width: 6

Background Intensity Spread: 16

Structuring Element Radius: 4

Greedy: 3

Fill Holes: 1

These parameters were determined manually by experimenting with the results CellTracer produced as the input parameters were manually varied. It is possible that this does not represent the optimal parameters for foreground background separation of these test images using CellTracer, however it does represent the best results we were able to obtain. In the CellTracer GUI, the left image displays the input grayscale image; the right image displays the current processing results. After background detection has been run, the green region denotes the background pixels that were found. From this point forward, the CellTracer algorithms will only be applied to the non-background (non-green) region, pixels that are labeled as background are ignored by any future processing steps.


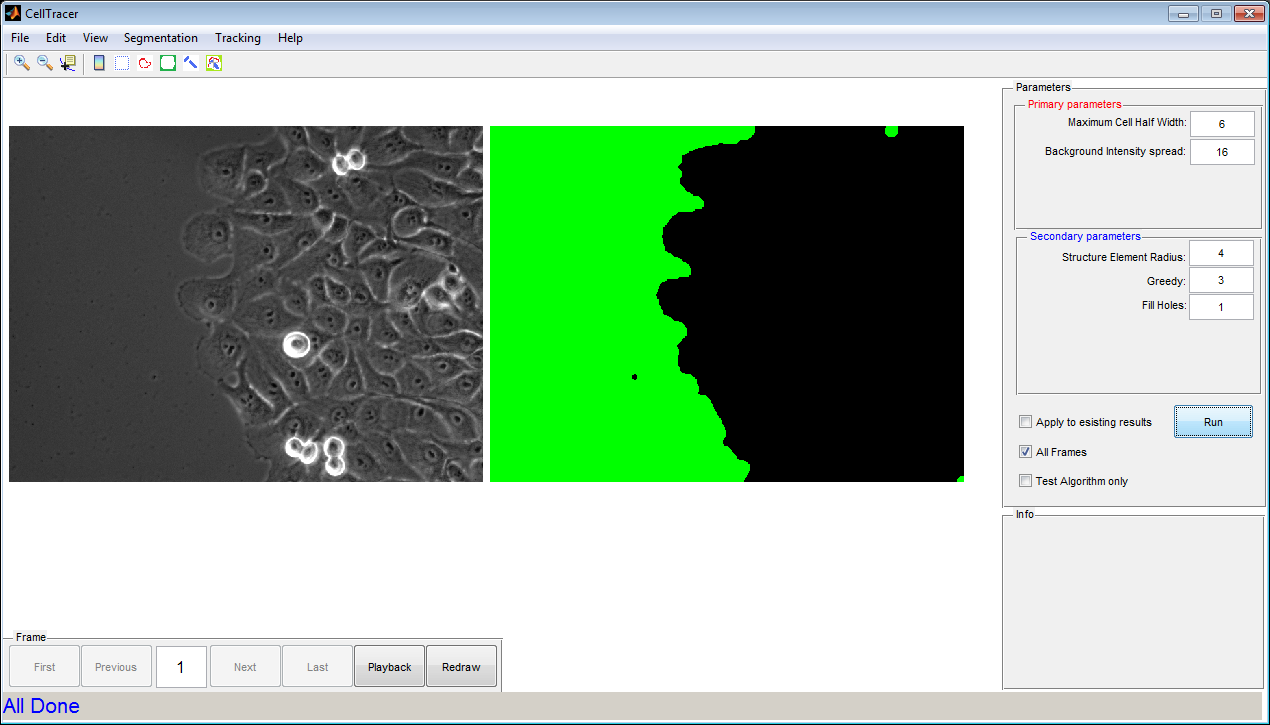


Figure 27: Foreground pixels extracted

The next step in segmenting single cells using CellTracer is to identify the borders between cells. For this dataset the best results were obtained by using the Threshold & Smoothing algorithm with the following parameters:

Maximum Cell Half Width: 30

Lower Ranking Threshold: 0.85

Upper Ranking Threshold: 1

Global Intensity Threshold: 255

Structure Element Radius: 5

The cell borders are shown in the CellTracer GUI windows as red regions.


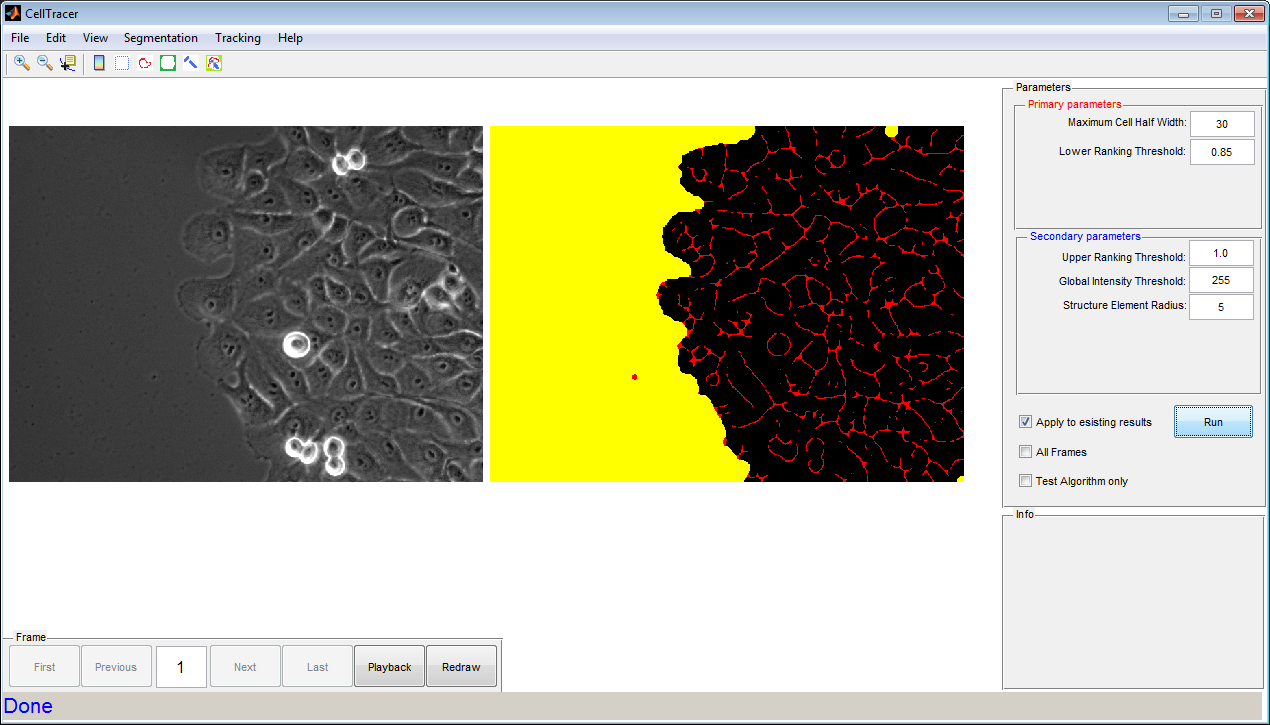


Figure 28: Cell Borders extracted using Threshold & Smoothing algorithm

The last step in single cell segmentation is to identify the cell objects using the information that has already been determined through the previous processing steps. For example the cell detection ignores pixels that are labeled as background, and the borders discovered in the previous step inform the cell separation. For this set of test images the Convex Model cell segmentation algorithm produced the best results with the following parameters:

Maximum Cell Score: 1

Minimum Cell Volume: 300

Structure Element Radius: 3

Smoothing Parameter: 0


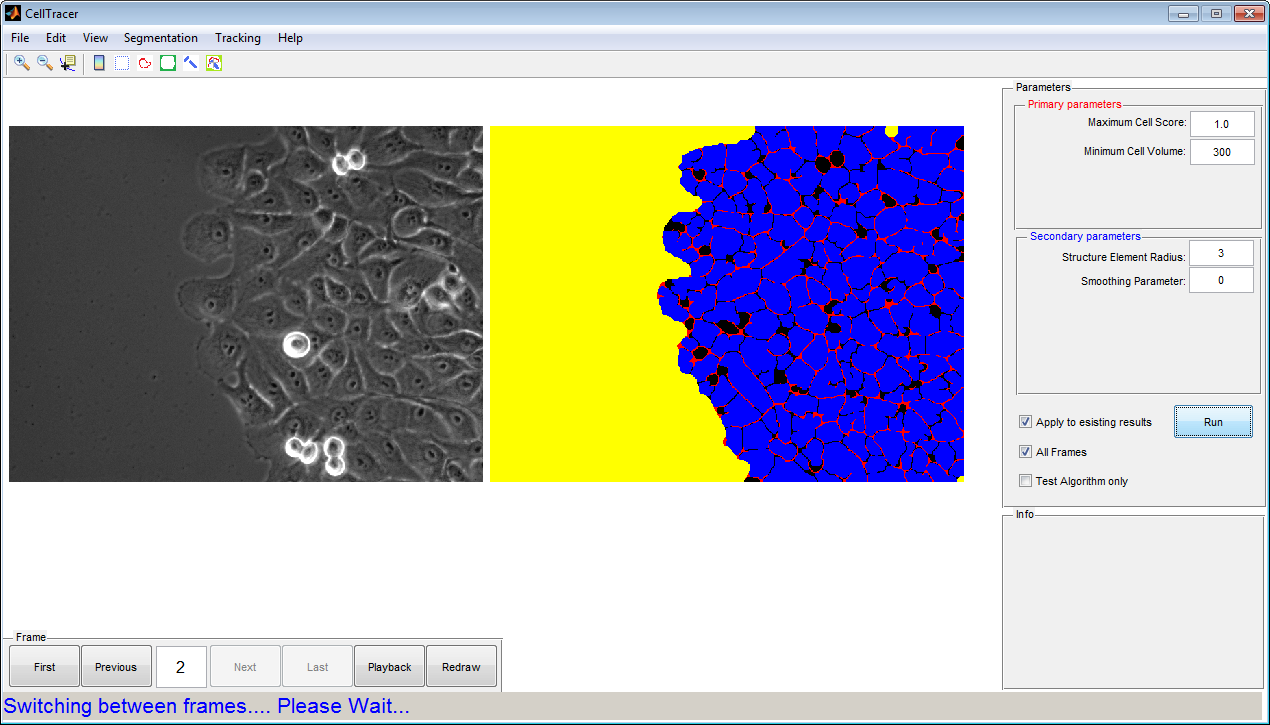


Figure 29: Result of the Convex Cell Model algorithm


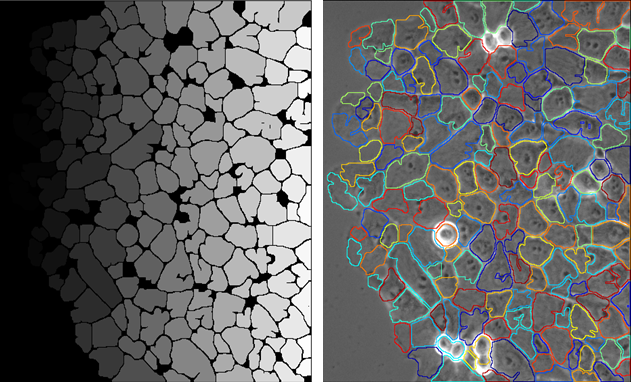


Figure 30: Resulting segmentation mask (left); Superimposed segmentation (right)


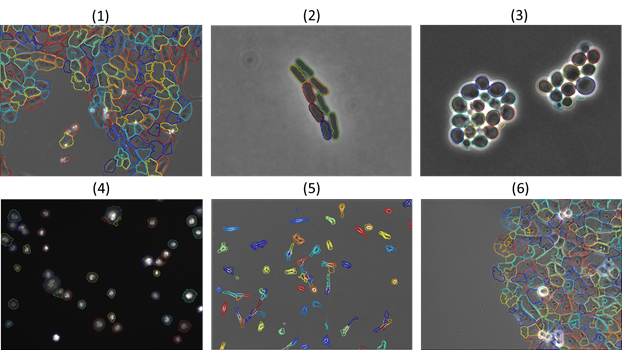


Figure 31: Example CellTracer Segmentation Results. (1) Bone Cancer Cells, (2) E.Coli Cells, (3) Yeast Cells, (4) A10 Cells, (5) 3T3 Cells (6) Breast Epithelial Cells

# SchnitzCells Pipeline

SchnitzCells is a collection of Matlab segmentation and tracking algorithms to automate cell segmentation available free of charge from <http://easerver.caltech.edu/wordpress/schnitzcells/>.

SchnitzCells contains two prepared segmentation pipelines. One to segment Bacillus cells, the other to segment E.Coli cells. These segmentation pipelines are accessed through the Matlab command line prompt. The test image sequence is loaded into SchnitzCells by calling the initschnitz function with the proper parameters for the given dataset.

p = initschnitz('VNmovie-01','2014-10-07','e.coli');

Phase contrast image segmentation is performed by calling p = segmoviephase(p), which performs the segmentation using the parameters defined using initschnitz. Fluorescent image segmentation is performed by calling p = segmoviefluor(p).

SchnitzCells has a detailed help manual and demonstration videos available on their webpage to explain the setup and use of their tools.

Both the E.Coli and Bacillus segmentation algorithms were tested on every dataset. The pipeline that produced the larger average ARI value across all image for that dataset was selected to represent SchnitzCells as the segmentation result for that dataset.


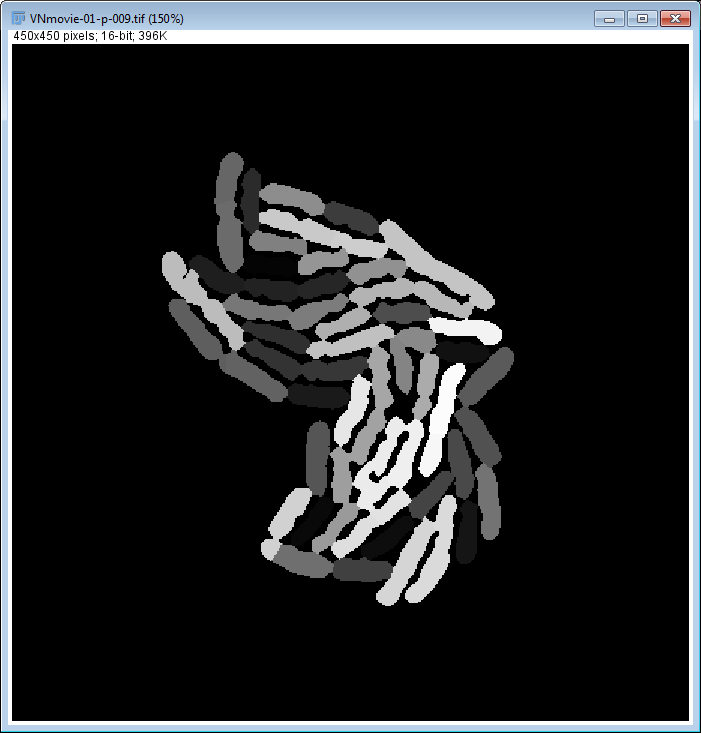

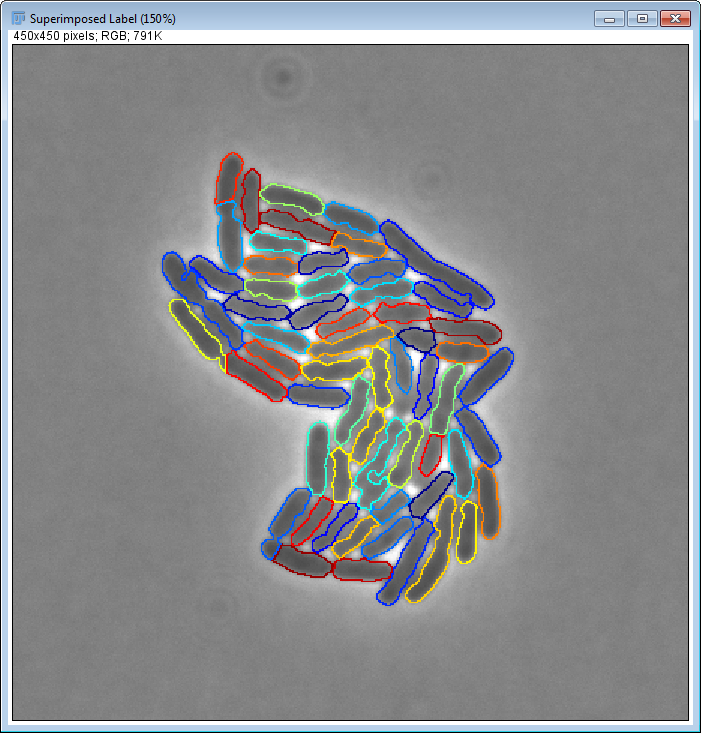


Figure 32: Example segmentation of an E.Coli image using SchnitzCells E.Coli segmentation pipeline


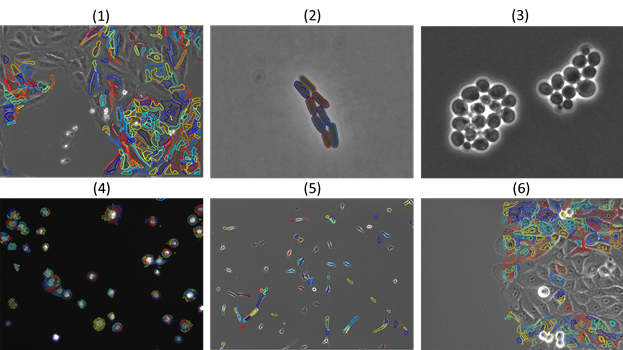


Figure 33: Example SchnitzCells Segmentation Results. (1) Bone Cancer Cells, (2) E.Coli Cells, (3) Yeast Cells, (4) A10 Cells, (5) 3T3 Cells (6) Breast Epithelial Cells

# Frlbm using level sets Pipeline

The inputs to this algorithm consist of the grayscale image to be segmented and a manually selected starting region for the contour. However, the resulting segmentation is very robust against changes to the starting region allowing the manual selection of this starting region to be replaced with an automated selection of a region in the middle of the image. The starting region was defined to be a 41x41 pixel square located at the center of the image.

The code the authors published with the paper is a Matlab script that segments a demonstration image that is packaged with the code. This script was converted into a function where the user passes in an image and a labeled segmented mask image is returned.

Figure 34: Segmentation result using FRLBM on a fluorescent image.


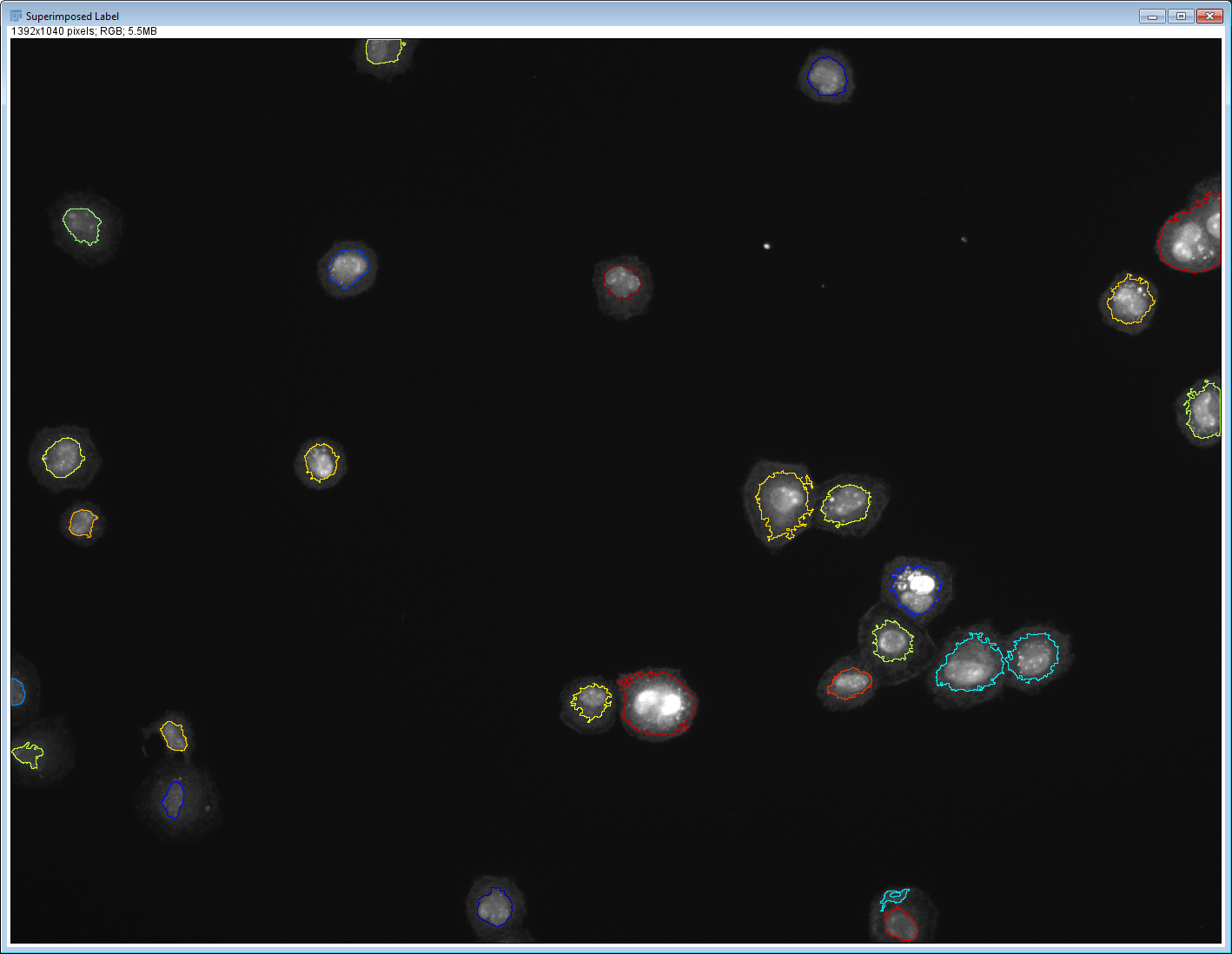

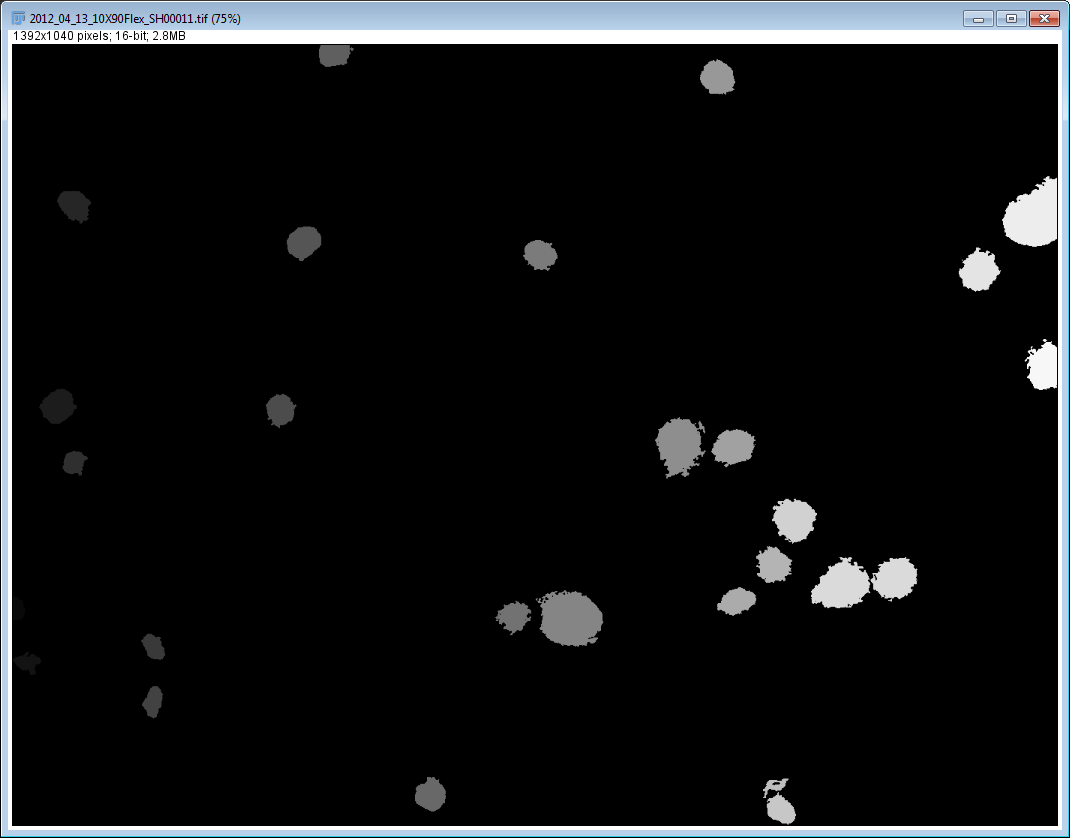


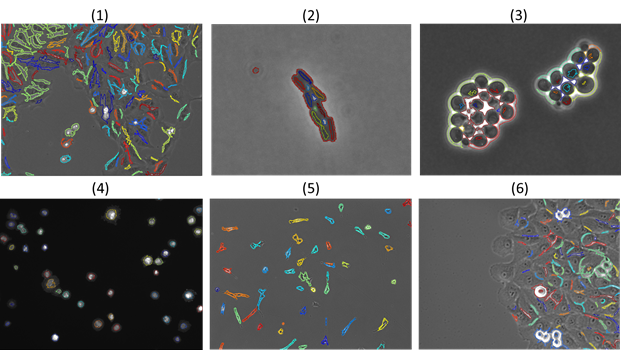


Figure 35: Example FRLBM Segmentation Results. (1) Bone Cancer Cells, (2) E.Coli Cells, (3) Yeast Cells, (4) A10 Cells, (5) 3T3 Cells (6) Breast Epithelial Cells

# Marker-Controlled Watershed (MCW) Pipeline

The Matlab code for the Marker-Controlled Watershed (MCW) segmentation was created by K. Parvati et al. 2008. The code is a Matlab function that takes as input the grayscale image to be segmented along with three segmentation parameters. The three parameters are two structuring element radii and a minimum object size in pixels.

To find the best parameters per image dataset being tested an exhaustive search was performed to find the set of input parameters that maximized the ARI value of the resulting segmentation. All three parameters were varied across reasonable ranges to produce the following optimal parameters per image dataset.

| Image Dataset | Strel Radius 1 | Strel Radius 2 | Min. Object Size |
| --- | --- | --- | --- |
| Bone Cancer Cells | 4 | 1 | 50 |
| E.Coli Cells | 6 | 4 | 10 |
| Yeast Cells | 2 | 1 | 20 |
| A10 Cells | 6 | 2 | 40 |
| 3T3 Cells | 4 | 2 | 40 |
| Breast Epithelial Cells | 4 | 2 | 70 |

The marker controlled watershed segmentation assigns every pixel to a labeled region. This does not reflect the reality of these image dataset which have background. To correct for this the largest connected component object in the segmented mask is considered background and set to zero.


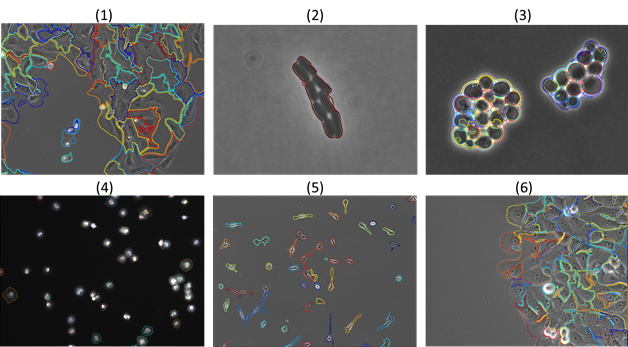


Figure 36: Example MCW Segmentation Results. (1) Bone Cancer Cells, (2) E.Coli Cells, (3) Yeast Cells, (4) A10 Cells, (5) 3T3 Cells (6) Breast Epithelial Cells

# Segmentation Results per Dataset

## Bone Cancer Cells


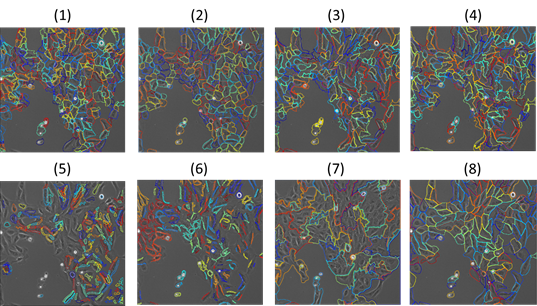


Figure 37: (1) CellProfiler based on region growing, (2) CellTracer, (3) FogBank, (4) FogBank wopg, (5) Schnitzcells, (6) Frlbm using level sets, (7) Marker-Controlled Watershed (MCW), and (8) Manual

## E.Coli Cells


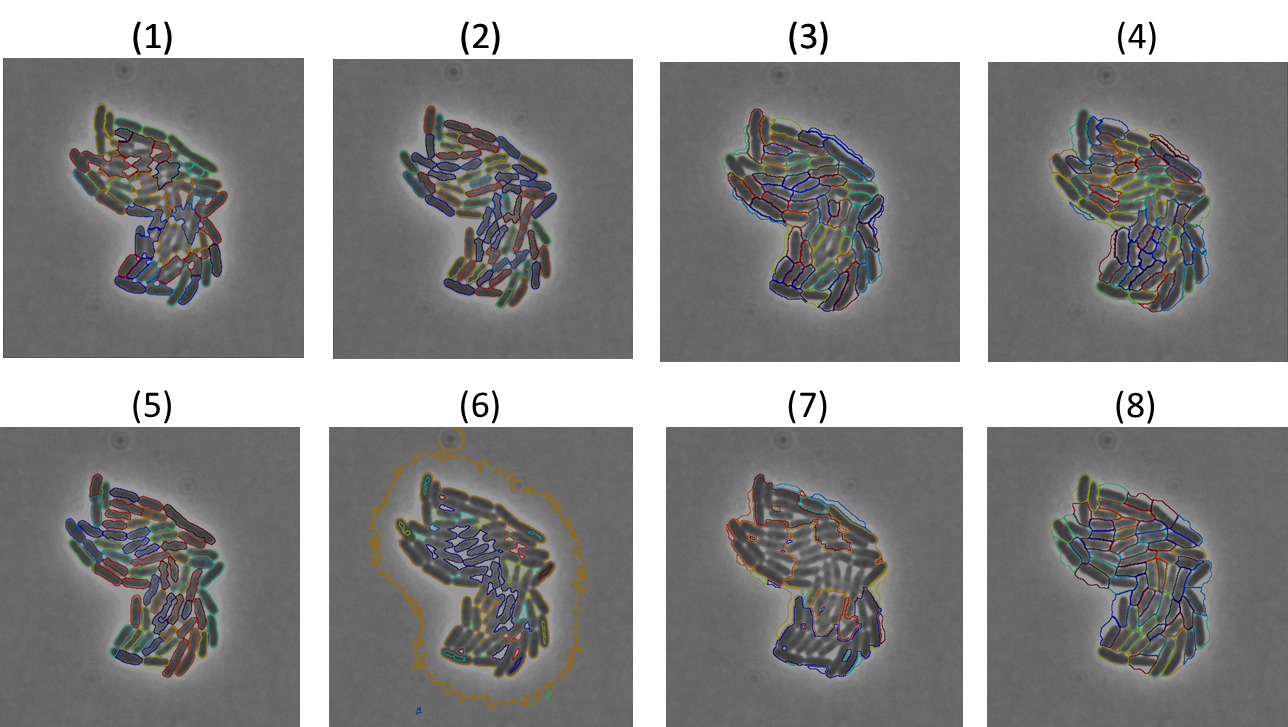


Figure 38: (1) CellProfiler based on region growing, (2) CellTracer, (3) FogBank, (4) FogBank wopg, (5) Schnitzcells, (6) Frlbm using level sets, (7) Marker-Controlled Watershed (MCW), and (8) Manual

## Yeast Cells


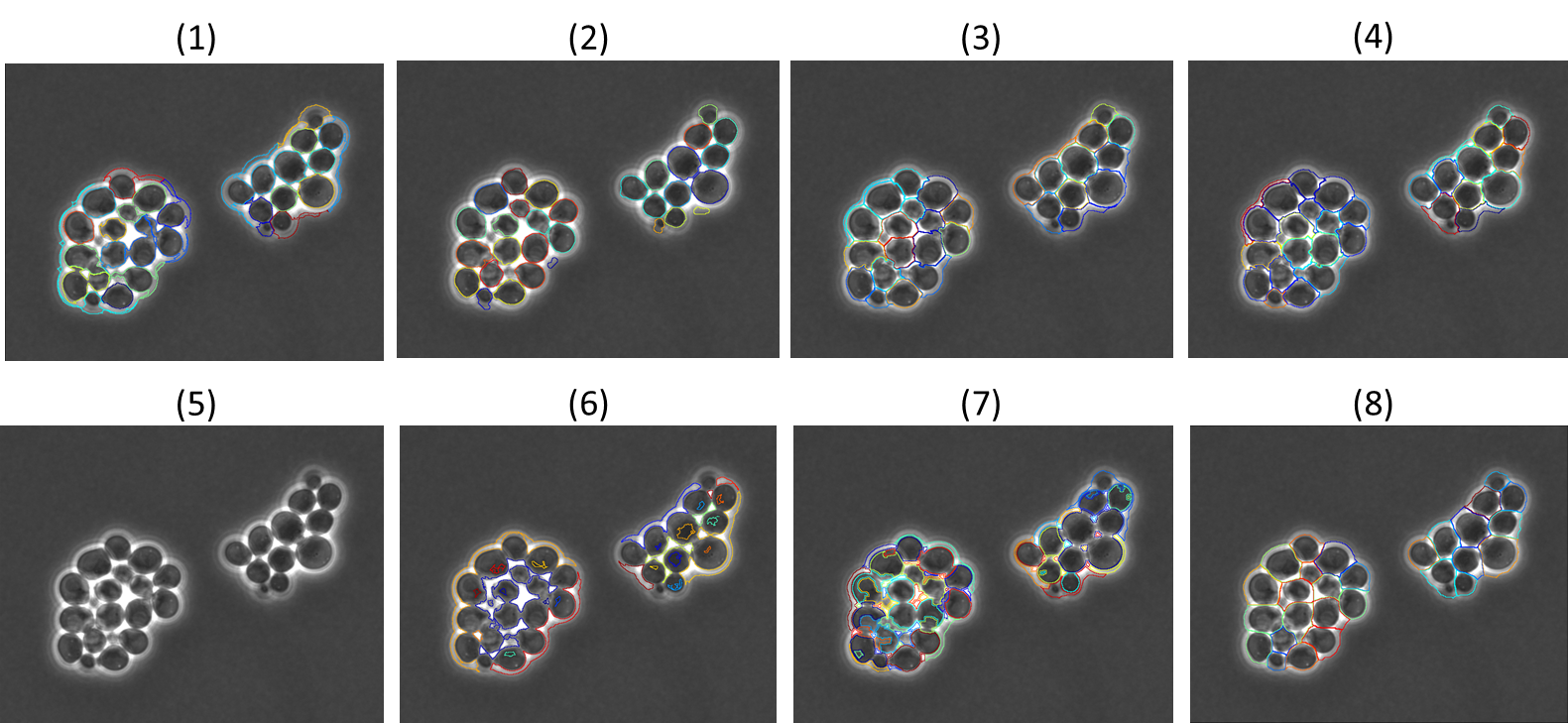


Figure 39: (1) CellProfiler based on region growing, (2) CellTracer, (3) FogBank, (4) FogBank wopg, (5) Schnitzcells, (6) Frlbm using level sets, (7) Marker-Controlled Watershed (MCW), and (8) Manual

## A10 Cells


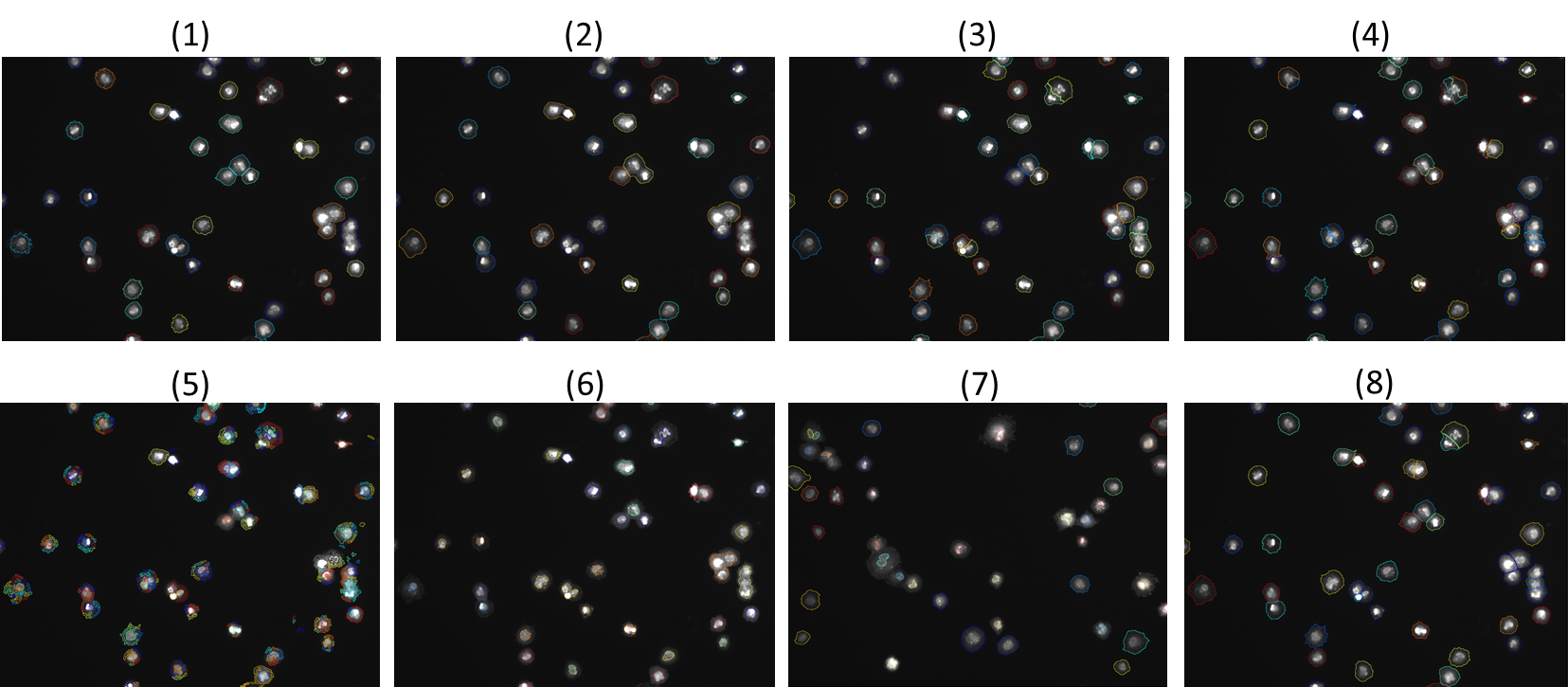


Figure 40: (1) CellProfiler based on region growing, (2) CellTracer, (3) FogBank, (4) FogBank wopg, (5) Schnitzcells, (6) Frlbm using level sets, (7) Marker-Controlled Watershed (MCW), and (8) Manual

## 3T3 Cells


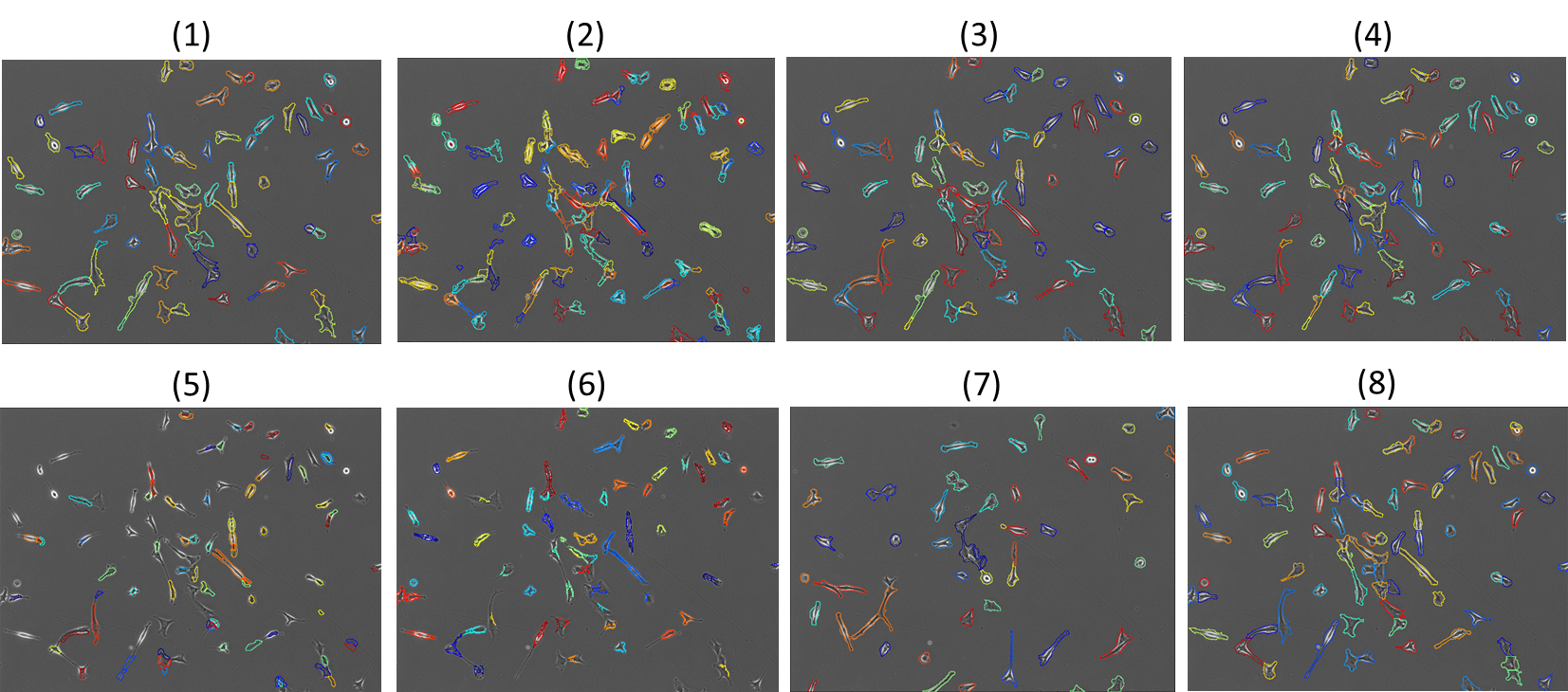


Figure 41: (1) CellProfiler based on region growing, (2) CellTracer, (3) FogBank, (4) FogBank wopg, (5) Schnitzcells, (6) Frlbm using level sets, (7) Marker-Controlled Watershed (MCW), and (8) Manual

## Breast Epithelial Cells


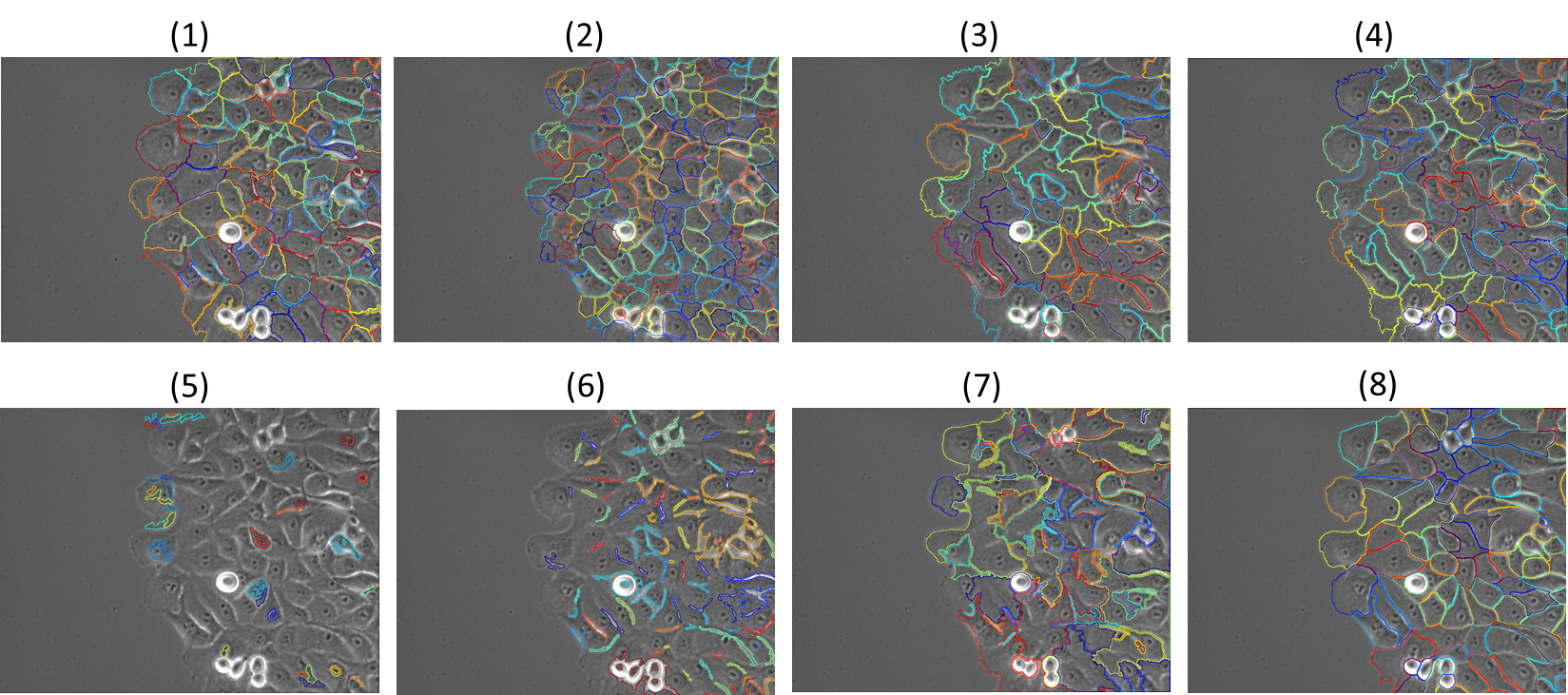


Figure 42: (1) CellProfiler based on region growing, (2) CellTracer, (3) FogBank, (4) FogBank wopg, (5) Schnitzcells, (6) Frlbm using level sets, (7) Marker-Controlled Watershed (MCW), and (8) Manual

1. Information Technology Laboratory, National Institute of Standards and Technology [↑](#footnote-ref-1)
2. Laboratory of Cellular and Molecular Biology, National Cancer institute [↑](#footnote-ref-2)
