# Supplementary material for: FogBank: a single cell segmentation across multiple cell lines and image modalities
Source: BMC Bioinformatics. 2014 Dec 30;15(1):431. doi: 10.1186/s12859-014-0431-x (PMC4301455; doi:10.1186/s12859-014-0431-x)
Supplement: Additional file 2: — Sensitivity analysis. This Additional file describes the sensitivity analysis performed on the input parameters of the FogBank technique over a breast epithelial sheet image. [file 12859_2014_431_MOESM2_ESM.docx]

FogBank: A Single Cell Segmentation across Multiple Cell Lines and Image Modalities

Joe Chalfoun^[[1]](#footnote-1)^, Mike Majurski^1^, Alden Dima^1^, Christina Stuelten^[[2]](#footnote-2)^, Adele Peskin^1^, and Mary Brady^1^

This Additional File describes the sensitivity analysis performed on the input parameters of the FogBank technique over a breast epithelial sheet image. There are 4 relevant parameters to analyze: P1 is the percentile threshold that defines the geodesic mask, P2 is the seed percentile threshold that defines the seed points from the lower intensity values, P3 is the seed minimum area size and P4 is the seed clustering distance.

The sensitivity analysis is done on a full factorial study on the entire range of each parameter (92213 parametric combinations). We then sorted the results in descending order with respect to the ARI value. We display the first 500 points on Figure 1. Over the top 500 combinations, ARI values change by as little as 4%, showing stability of the analysis as a function of all 4 parameters. Almost the same accuracy is achieved for a border threshold higher than 90^th^ percentile, a seed threshold less than the 3^rd^ percentile, a minimum seed size between 3 and 10 and a clustering distance between 10 and 20.

Figure 1: Sensitivity plot for FogBank parameters

1. Information Technology Laboratory, National Institute of Standards and Technology [↑](#footnote-ref-1)
2. Laboratory of Cellular and Molecular Biology, National Cancer institute [↑](#footnote-ref-2)
