# Supplementary material for: FogBank: a single cell segmentation across multiple cell lines and image modalities
Source: BMC Bioinformatics. 2014 Dec 30;15(1):431. doi: 10.1186/s12859-014-0431-x (PMC4301455; doi:10.1186/s12859-014-0431-x)
Supplement: Additional file 3: — Detailed description of the manual reference dataset segmentation. This Additional file describes in detail the creation of the reference datasets. We describe the step by step creation of the 6 manually segmented datasets by expert scientists. These masks are used to quantify the performance of the Fog Bank segmentation. [file 12859_2014_431_MOESM3_ESM.docx]

FogBank: A Single Cell Segmentation across Multiple Cell Lines and Image Modalities

Joe Chalfoun^[[1]](#footnote-1)^, Mike Majurski^1^, Alden Dima^1^, Christina Stuelten^[[2]](#footnote-2)^, Adele Peskin^1^, and Mary Brady^1^

This Additional file describes in detail the creation of the reference datasets. We describe the step by step creation of the manually segmented datasets by expert scientists. These masks are used to quantify the performance of the FogBank segmentation.

# Raw Images

We used six datasets to create manual segmentation: (1) 10 phase images of bone cancer cells from Broad Institute [1] with a total of 2168 manually detected cells, (2) 10 Fluorescent images of E. coli cells from Duke University [2][3] with a total of 237 manually detected cells, (3) 10 Fluorescent images of yeast cells from Duke University [2][3] with a total of 153 manually detected cells, (4) 10 Fluorescent A10 rat cells from National Institute of Standard and Technology (NIST) with a total of 347 manually detected cells, (2) 10 phase images of NIH 3T3 cells from NIST with a total of 656 manually detected cells, and (1) 59 phase images of breast epithelial sheets from NIH with a total of 5722 manually detected cells. This dataset is available for download from <https://isg.nist.gov/>.

# Reference dataset Step-by-step Creation

Manual segmentation of all six reference datasets is done with the same methodology. We display this methodology on breast epithelial cell sheets. An expert scientist segmented these images using ImageJ [4], contouring the cell edges by the pencil tool with pixel value of zero. The expert worked on the raw phase images, tracing all cell boundaries with the pencil tool in ImageJ, leaving 1 pixel as background between cell edges in an 8 connected neighborhood. The results are shown in Figure 1. The masks are created by converting the outlines to binary in ImageJ and then filling the holes as shown in Figure 2.

Figure 1- (Left) Manual cell separation on phase contrast image. (Right) zoomed image to show the manual boundaries that have been highlighted with the pencil tool in ImageJ.


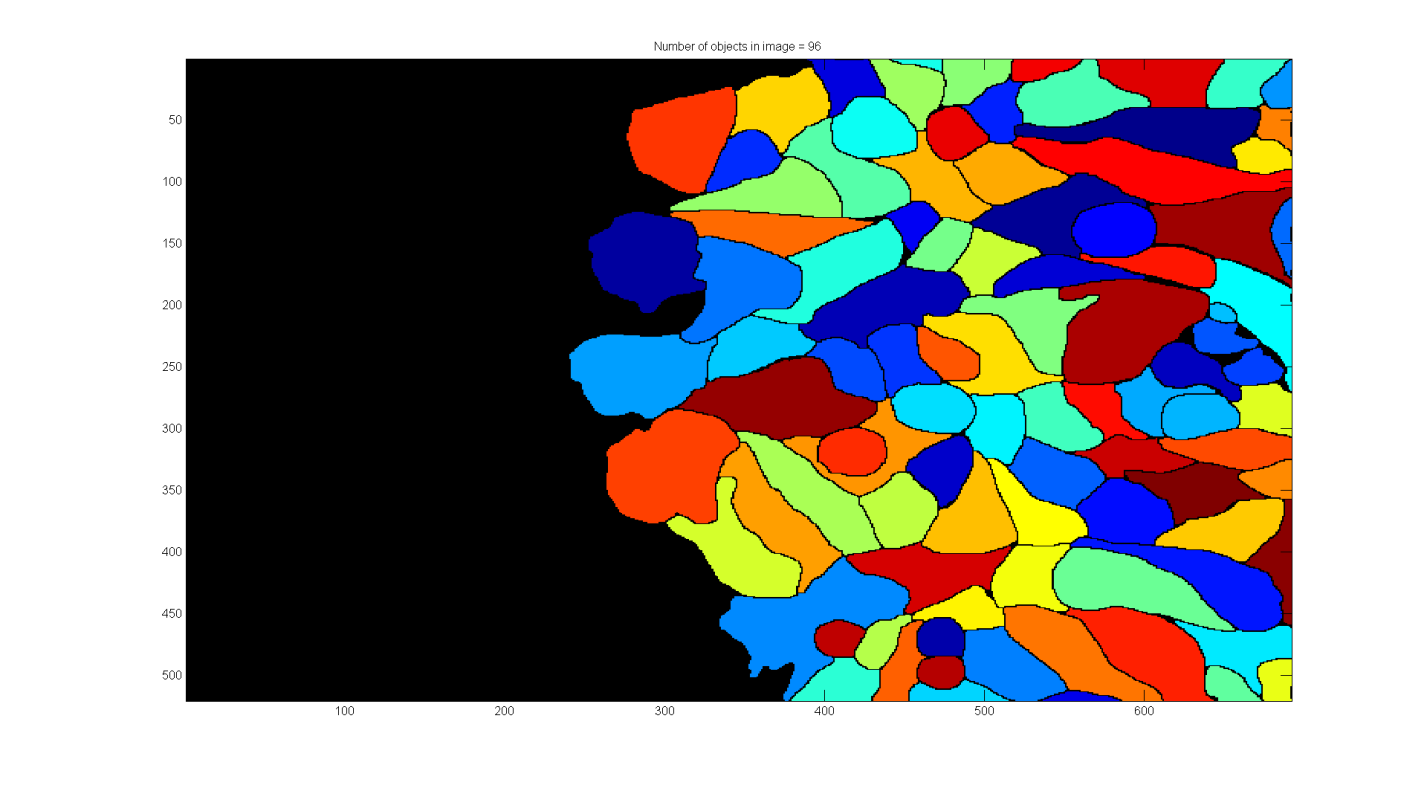


Figure 2- Labeled mask generated from the phase image after converting to binary mask, filling the holes and applying pixel connectivity algorithm. The colors are random and used only for display purposes.

# References

[1] I. a Khan, M. Lupi, L. Campbell, S. C. Chappell, M. R. Brown, M. Wiltshire, P. J. Smith, P. Ubezio, and R. J. Errington, “Interoperability of time series cytometric data: a cross platform approach for modeling tumor heterogeneity.,” *Cytometry. A*, vol. 79, no. 3, pp. 214–26, Mar. 2011.

[2] Q. Wang, J. Niemi, C.-M. Tan, L. You, and M. West, “Image segmentation and dynamic lineage analysis in single-cell fluorescence microscopy.,” *Cytometry. A*, vol. 77, no. 1, pp. 101–10, Jan. 2010.

[3] N. Rosenfeld, T. J. Perkins, U. Alon, M. B. Elowitz, and P. S. Swain, “A fluctuation method to quantify in vivo fluorescence data.,” *Biophys. J.*, vol. 91, no. 2, pp. 759–66, Jul. 2006.

[4] J. Schindelin, I. Arganda-Carreras, E. Frise, V. Kaynig, M. Longair, T. Pietzsch, S. Preibisch, C. Rueden, S. Saalfeld, B. Schmid, J.-Y. Tinevez, D. J. White, V. Hartenstein, K. Eliceiri, P. Tomancak, and A. Cardona, “Fiji: an open-source platform for biological-image analysis.,” *Nat. Methods*, vol. 9, no. 7, pp. 676–82, Jul. 2012.

1. Information Technology Laboratory, National Institute of Standards and Technology [↑](#footnote-ref-1)
2. Laboratory of Cellular and Molecular Biology, National Cancer institute [↑](#footnote-ref-2)
